# Combination of RUNX1 inhibitor and gemcitabine mitigates chemo‐resistance in pancreatic ductal adenocarcinoma by modulating BiP/PERK/eIF2α-axis-mediated endoplasmic reticulum stress

**DOI:** 10.1186/s13046-023-02814-x

**Published:** 2023-09-11

**Authors:** Chunhua She, Chao Wu, Weihua Guo, Yongjie Xie, Shouyi Li, Weishuai Liu, Chao Xu, Hui Li, Pei Cao, Yanfang Yang, Xiuchao Wang, Antao Chang, Yukuan Feng, Jihui Hao

**Affiliations:** 1 Department of Neurosurgery and Neuro-Oncology, Tianjin Medical University Cancer Institute and Hospital, National Clinical Research Center for Cancer; Key Laboratory of Cancer Prevention and Therapy, Tianjin’s Clinical Research Center for Cancer, Tianjin, 300060 China; 2 Department of Pancreatic Cancer, Tianjin Medical University Cancer Institute and Hospital, National Clinical Research Center for Cancer; Key Laboratory of Cancer Prevention and Therapy, Tianjin’s Clinical Research Center for Cancer, Tianjin, 300060 China; 3 Department of Pain Management, Tianjin Medical University Cancer Institute and Hospital, National Clinical Research Center for Cancer, Key Laboratory of Cancer Prevention and Therapy, Tianjin, 300060 China; 4 School of Medicine, Nankai University, Tianjin, 300060 China; 5 Second Department of Breast Cancer, Tianjin Medical University Cancer Institute and Hospital, National Clinical Research Center for Cancer, Key Laboratory of Cancer Prevention and Therapy, Tianjin’s Clinical Research Center for Cancer, Key Laboratory of Breast Cancer Prevention and Therapy, Tianjin Medical University, Ministry of Education, Tianjin, 300060 China; 6 Mudanjiang Medical University, Mudanjiang, 157011 China

**Keywords:** RUNX1, Gemcitabine resistance, ER stress, BiP, PDAC

## Abstract

**Background:**

Gemcitabine (GEM)-based chemotherapy is the first-line option for pancreatic ductal adenocarcinoma (PDAC). However, the development of drug resistance limits its efficacy, and the specific mechanisms remain largely unknown. RUNX1, a key transcription factor in hematopoiesis, also involved in the malignant progression of PDAC, but was unclear in the chemoresistance of PDAC.

**Methods:**

Comparative analysis was performed to screen GEM-resistance related genes using our single-cell RNA sequencing(scRNA-seq) data and two public RNA-sequencing datasets (GSE223463, GSE183795) for PDAC. The expression of RUNX1 in PDAC tissues was detected by qRT-PCR, immunohistochemistry (IHC) and western blot. The clinical significance of RUNX1 in PDAC was determined by single-or multivariate analysis and survival analysis. We constructed the stably expressing cell lines with shRUNX1 and RUNX1, and successfully established GEM-resistant cell line. The role of RUNX1 in GEM resistance was determined by CCK8 assay, plate colony formation assay and apoptosis analysis in vitro and in vivo. To explore the mechanism, we performed bioinformatic analysis using the scRNA-seq data to screen for the endoplasm reticulum (ER) stress signaling that was indispensable for RUNX1 in GEM resistance. We observed the cell morphology in ER stress by transmission electron microscopy and validated RUNX1 in gemcitabine resistance depended on the BiP/PERK/eIF2α pathway by in vitro and in vivo oncogenic experiments, using ER stress inhibitor(4-PBA) and PERK inhibitor (GSK2606414). The correlation between RUNX1 and BiP expression was assessed using the scRNA-seq data and TCGA dataset, and validated by RT-PCR, immunostaining and western blot. The mechanism of RUNX1 regulation of BiP was confirmed by ChIP-PCR and dual luciferase assay. Finally, the effect of RUNX1 inhibitor on PDAC was conducted in vivo mouse models, including subcutaneous xenograft and patient-derived xenograft (PDX) mouse models.

**Results:**

RUNX1 was aberrant high expressed in PDAC and closely associated with GEM resistance. Silencing of RUNX1 could attenuate resistance in GEM-resistant cell line, and its inhibitor Ro5-3335 displayed an enhanced effect in inhibiting tumor growth, combined with GEM treatment, in PDX mouse models and GEM-resistant xenografts. In detail, forced expression of RUNX1 in PDAC cells suppressed apoptosis induced by GEM exposure, which was reversed by the ER stress inhibitor 4-PBA and PERK phosphorylation inhibitor GSK2606414. RUNX1 modulation of ER stress signaling mediated GEM resistance was supported by the analysis of scRNA-seq data. Consistently, silencing of RUNX1 strongly inhibited the GEM-induced activation of BiP and PERK/eIF2α signaling, one of the major pathways involved in ER stress. It was identified that RUNX1 directly bound to the promoter region of BiP, a primary ER stress sensor, and stimulated BiP expression to enhance the reserve capacity for cell adaptation, which in turn facilitated GEM resistance in PDAC cells.

**Conclusions:**

This study identifies RUNX1 as a predictive biomarker for response to GEM-based chemotherapy. RUNX1 inhibition may represent an effective strategy for overcoming GEM resistance in PDAC cells.

**Supplementary Information:**

The online version contains supplementary material available at 10.1186/s13046-023-02814-x.

## Background

Pancreatic ductal adenocarcinoma (PDAC) is a highly lethal disease with an average 5-year survival rate of less than 10% [1]. Surgical resection is regarded as the only potentially curative treatment, but only 10–20% of patients with PDAC present with resectable disease at the time of diagnosis because most patients remain asymptomatic until the disease reaches an advanced stage [2]. Consequently, chemotherapy, including adjuvant chemotherapy with gemcitabine (GEM) after surgery, FOLFIRINOX chemotherapy, and GEM plus nanoparticle albumin-bound paclitaxel, remains the best treatment option for patients who are not surgical candidates [3]. In summary, over the past two decades, GEM has remained the primary drug of choice for PDAC therapy, with a significant effect on patient survival.

Despite the widespread use of GEM, primary and acquired drug resistance is one of the main obstacles encountered in GEM-based chemotherapy, and the underlying mechanisms remain poorly understood [4]. Among multiple factors responsible for GEM resistance in PDAC, drug-induced endoplasmic reticulum (ER) stress has recently been regarded as a major hindrance to successful chemotherapy [5]. ER stress, which can be initiated by various stimuli, including hypoxia, glucose deprivation, oxidative stress, and drugs, is a cytoprotective pathway to maintain cell homeostasis, release stress from exogenous or endogenous factors, and confer drug resistance in various cancers [6]. Under ER stress, the binding immunoglobulin protein (BiP), a ER-resident molecular chaperone, prefers to bind to misfolded or unfolded proteins owing to its higher affinity binding for them, and is therefore titrated away from three ER transmembrane proteins: PKR-like endoplasmic reticulum kinase (PERK), the inositol-requiring protein 1α (IRE1α) and activating transcription factor 6 (ATF6), which initiate the unfolding protein response (UPR) [7]. However, BiP binds to these proteins to restrain their activation under normal condition. It has been suggested that BiP functions as primary sensor in the UPR activation, and coordinates with PERK, IRE1α and ATF6 signaling to restore ER homeostasis. In general, BiP is geared towards sensing mis-folded proteins, and once BiP is bound to mis-folded proteins, it engenders an allosteric change that alters its affinity for IRE1α and PERK, causing their release from the complex, a process that is suggested to be coupled to UPR activation [8]. As the key ER stress sensor in the activation of the UPR pathway, the regulated factors affect BiP abundance, and the role of the BiP/PERK/eIF2 α -axis-mediated ER stress in GEM resistance requires further investigation.

The RUNX1 transcription factor plays multifaceted functions in hematopoietic diseases and solid cancers, behaving in a context-dependent manner [9, 10]. Under hypoxic stress, RUNX1 is activated to decelerate the cell cycle of oligodendrocyte precursor cells, facilitating the quiescence of adult somatic stem cells [11]. RUNX1 also modulates the biosynthetic activity to adapt to the genotoxic stress, and its deficiency provided a selective advantage for certain types of hematopoietic stem and progenitor cells (HSPCs) [12]. Recently, correlation of RUNX1 with ER stress was reported in the formation of neurofibromagenesis [13]. It is determined that RUNX1 enhances Schwann cells adaption to ER stress in virtue of its transcriptionally roles in ribosome gene expression. However, the modulation mechanism of RUNX1 on ER stress in tumor cells has not been reported yet. Though the RUNX family has been more widely known and identified as oncogenes involved in PDAC metastasis [14], the versatile roles of RUNX1 in PDAC has not been fully elucidated. It is noteworthy that several studies have identified targeting RUNX1 can result in promising therapeutic effects against various diseases [15]. Hence, as an attractive target, RUNX1 holds great potential for mitigating the ER stress-mediated GEM resistance.

Our findings confirm the onco-supportive role of RUNX1 in the malignant progression of PDAC. We first present evidence of RUNX1 modulation on ER stress and demonstrate that RUNX1 binds to the promoter region of BiP and transcriptionally promotes BiP expression, adapting to ER stress by activating the PERK/eIF2α pathway. Importantly, silencing RUNX1 reverses GEM resistance in GEM-resistant cell line, and its inhibitor Ro5-3335 enhances the antitumor activity of GEM in patient-derived-xenograft (PDX) mouse models and GEM-resistant xenografts. Therefore, RUNX1 inhibition could be a potential combination therapy for overcoming GEM resistance in PDAC.

## Methods

### Cell culture and reagents

Human PDAC cell lines CFPAC-1, SW1990, and BxPC3 were purchased from the Committee of Type Culture Collection of the Chinese Academy of Sciences (Shanghai, China), and L3.7–2 and MIA-PaCa2 were obtained from the American Type Culture Collection (ATCC). SW1990, BxPC3, and L3.7–2 cells were cultured in Roswell Park Memorial Institute Medium (RPMI) 1640 containing 10% fetal bovine serum (FBS) and 1% penicillin/streptomycin (P/S). MIA-PaCa2 cells were cultured in Dulbecco’s modified Eagle’s medium (DMEM) supplemented with 10% FBS and 1% P/S. All cells were incubated at 37 °C and 5% CO_2_ in humidified air.

The reagents used in the experiments were as follow: Ro5-3335 (#4694, TOCRIS), gemcitabine (#1,288,463, Sigma-Aldrich, St. Louis, MO), sodium phenylbutyrate (4-PBA, #1716–12-7, Sigma-Aldrich, St. Louis, MO), thapsigargin (S7895, Selleck) and GSK2606414 (#1,337,531–36-8, MedChemExpress).

### Tissue samples and immunohistochemistry (IHC) assay

Tumor tissues were obtained from patients who underwent radical surgery between December 2011 and December 2017 at the Tianjin Medical University Cancer Institute and Hospital (Tianjin, China). Patient and mouse tumor tissues were fixed in formalin and embedded in paraffin wax. The 4 μm microsections were stained with antibodies of RUNX1, BiP, p-eIF2α, and caspase 3 using a DAB substrate kit (Maixin Biotech, Fuzhou, China). The results were reviewed by two pathologists blinded to the clinicopathological data. Staining was scored by multiplying the intensity (0, negative; 1, low; 2, medium; 3, high) and staining area (0, no staining; 1: 1–25% stained; 2:26–50% stained; 3:51–100% stained). The final score was graded as follows: 0, negative; 1–3, low staining ( +); 4–6, medium staining (+ +); and > 6, high staining (+ + +). The association between RUNX1 expression and clinical features, such as age, sex, tumor stage, and histological grade was evaluated using Pearson's correlation analysis and logistic regression models. The results are presented as hazard ratios (HR) with 95% confidence intervals (CIs). All patients signed a consent form for the use of their specimens and information for research, which was approved by the Ethics Committee of the Tianjin Medical University Cancer Institute and Hospital.

### Transient transfection and lentivirus infection

Small interfering RNA (siRNA) for targeted genes (RUNX1 and BiP) were purchased from Gene Pharma (Shanghai, China). Before transfection, PDAC cells were seeded in six-well plates at a density of 5 × 10^5^ cells/well. When the cells reached 70% confluence, the siRNA complexes were added to each well and incubated with Lipofectamine 2000 (Invitrogen) for 48 h. The cells were then collected for subsequent experiments.

The sequences of siRNA targeting RUNX1(from 5’ to 3’):

- #1: GGAUCCAUUGCCUCUCCUUTT; AAGGAGAGGCAAUCCAUCCTT
- #2: GGAUACAAGGCAGAUCCAATT; UUGGAUCUGCCUUGUAUCCTT
  The sequences of siRNA targeting BiP(from 5’ to 3’):
- #1: AGUGUUGGAAGAUUCUGAU; UCACAACCUUCUAAGACUA
- #2: GGAGCGCAUUGAUACUAGA; CCUCGCGUAACUAUGAUCU

For gene expression analysis, viral supernatants were purchased from SyngenTech (Beijing, China). For stable knockdown, lentivirus-shRUNX1 and a negative control were constructed by GenePharma (Shanghai, China). CFPAC1, L3.7–2, SW1990, and MIA-PaCa2 were incubated for 24 h with the virus supernatants containing 8 μg/mL polybrene, and then cultured for 1 week with fresh culture media containing puromycin (1 µg/mL) without P/S. After validation by qRT-PCR and western blotting, selected cells were propagated and used for further experiments.

### Cell viability assay

Cell viability was evaluated with a cell counting kit 8 (CCK-8; Sigma-Aldrich). Briefly, cells were seeded into a 96-well plate at 5000 cells per well. After 24 h, cells were treated with different concentrations of gemcitabine. At the indicated time points, fresh medium containing 10% CCK-8 was added to each well instead of the primary medium. After incubation for 4 h at 37 °C, the OD value at 450 nm was read using a plate reader.

### Cell apoptosis assay

Apoptosis was measured by flow cytometry using APC and propidium iodide (PI; Invitrogen, USA). Cells were seeded in 6-well plates and treated with 2µ M GEM. After 48 h, the cells were harvested and stained with APC and PI, according to the manufacturer’s instructions. The cells were immediately analyzed using a FACSAria flow cytometer (BD Biosciences).

### TUNEL assay

The TUNEL assay was performed according to the instructions of the in situ cell death detection kit. Briefly, after dewaxing and hydration, slides were immersed in 0.5% triton x-100, followed by staining with TUNEL and DAPI solutions for the indicated times. The images were captured using a fluorescence microscope. The average percentage of positive cells in five fields was calculated.

### Plate colony formation assay

Cells were cultured in 6-well plates at 1000 cells/well for 2 weeks, and fresh medium was added every 3 days. At the end of the experiment, the medium was removed and colonies were fixed with 4% paraformaldehyde (w/v), stained with (0.1% w/v) crystal violet, and then counted using Image J software.

### Western blot

Cells were lysed using RIPA buffer containing a proteinase inhibitor (Sigma-Aldrich, USA) and phosphatase inhibitor cocktail (Bimake, USA). A Pierce BCA protein assay kit (Thermo Fisher Scientific, USA) was used to measure the protein concentration. Equal amounts of protein (30 ug) were separated by sodium dodecyl sulfate–polyacrylamide gel (SDS-PAGE) electrophoresis, transferred onto a PVDF membrane and then detected by chemiluminescence. Antibodies used for Western blot were as follow: RUNX1 (ab23980, Abcam), RUNX1 (ab189172, Abcam), BiP (ab21685, Abcam), p-IRE1α (ab48187,S724, Abcam), ATF6 (ab227830, Abcam), XBP-1(sc-8015, Santa cruz) and the antibodies from Cell Signaling Tech were PERK(#5683), phospho-PERK(#3179), IRE1α(#3294), eIF2α(#5324), phospho-eIF2α(#3398,ser 51), ATF4(#11815), Caspase-3(#9662), GAPDH (#5174).

### RNA extraction and quantitative polymerase chain reaction (qPCR)

Total RNA was extracted using TRIzol reagent (Invitrogen, USA), and first-strand cDNA was synthesized using a first-strand synthesis system (Takara, Japan) for reverse transcription (RT). cDNA levels were analyzed by qPCR. Each experiment was independently performed in triplicate. The primers used for qPCR listed in Supplementary Table 1.

### Transmission electron microscopy image (TEM)

TEM was performed at the Department of Electron Microscopy at the Institute of Hematology and Blood Diseases Hospital, Chinese Academy of Medical Sciences, and Peking Union Medical College. The cells were harvested and fixed in a 2.5% glutaraldehyde solution for 24 h. The samples were then examined using the TEM facility electron microscope for subsequent analysis.

### Chromatin immunoprecipitation (ChIP) assay

The ChIP Assay was conducted using a ChIP kit (Millipore) according to the manufacturer’s instructions. PCR was performed using primers spanning two regions identified in the promoter region of human BiP. PCR products were analyzed by 1% agarose gel electrophoresis.

### Luciferase assays

Luciferase activity was tested in transfected cells using a dual-luciferase reporter assay system (Promega, USA). Briefly, sw1990-RUNX1 and SW1990-vector cells were seeded at a density of 5 × 10^5^ cells/well in 24-well plates. At 80% confluence, the cells were transiently transfected with the pGL3-BiP promoter wild-type (wt), pGL3-BiP promoter mutant (-326- -325, CG → AA), or pGL3-control vector. 24 h after transfection, luciferase assays were performed at room temperature, according to the manufacturer’s instructions. The data are presented as fold-changes relative to those cells transfected with the control vectors after normalization to Renilla activity.

### Single-cell RNA sequencing (scRNA-seq) data and analysis

The scRNA-seq data used in this study were obtained from two public datasets: HRA000433 (14 patients with PDAC) downloaded from The Genome Sequence Archive for Human (GSA-Human) and CRA001160 (24 patients with PDAC and 11 control pancreatic tissues) obtained from the Genome Sequence Archive under project PRJCA001063. Raw sequencing data were processed using the Cell Ranger pipeline and filtered to exclude cells with low quality or low read counts. Raw sequencing reads were filtered for low-quality bases and adaptor sequences using the Trimmomatic software. The resulting high-quality reads were mapped to the reference genome using STAR software, and gene expression was quantified using featureCounts. Cells with fewer than 200 detected genes or with mitochondrial gene expression greater than 25% were excluded from downstream analysis. Cell clustering was performed using the Seurat package (Rstudio 4.2.1). Briefly, the cells were first normalized and log-transformed using the SCTransform method. Principal component analysis (PCA) was performed on the top variable genes, and the first 30 principal components were used for clustering analysis using the FindClusters function at a resolution of 0.5. A t-SNE plot was generated to visualize the cell clusters, and differential gene expression analysis was performed using the FindMarkers function. The results of the single-cell transcriptome data analysis were visualized using various tools and software, such as t-SNE plots and heatmaps, to provide clear and concise data representation.

### Bioinformatic processing of The Cancer Genome Atlas (TCGA) data

Transcriptome and clinical data of patients with pancreatic cancer were obtained from TCGA database. RNA-seq data were downloaded from the Genomic Data Commons (GDC) portal, and clinical data were obtained from the TCGA Data Portal. Raw sequencing reads were preprocessed using the GDC RNA-seq pipeline, which included adaptor trimming, quality control, and alignment to the reference genome (GRCh38) using STAR software. Gene expression was quantified using feature counts and the resulting count matrix was normalized using the trimmed mean of M-values (TMM) method. Clinical data were preprocessed and filtered to exclude patients with missing information or inadequate follow-up. Differential gene expression analyses were performed using EdgeR package. Briefly, the count matrix was filtered to remove low-expression genes and the remaining genes were tested for differential expression using the exact test. Differentially expressed genes (DEGs) were identified based on a false discovery rate (FDR) cutoff of 0.05 and a log2 fold change threshold of 1.5. The results of the transcriptome and clinical data analyses were visualized using various tools and software, such as survival curves and scatterplots, to provide a clear and concise data representation.

### Establishment of gemcitabine-resistant cell model

When cells grew at 80%-90% confluence, gemcitabine at a low concentration (1/10 of the IC50 of the BxPC3 cell line) was added for treatment and cultured in cell incubator. when the cell density reached 50%, the culture medium was discarded, washed by PBS for 2 times, and replaced with drug-free medium to continue the culture. Repeat the above drug treatment six times when the cell growth density returns to 80%-90% again. After the cells grew stably at this concentration, sequentially increase the drug concentration (added by double) and treat in the same way until the cells can grow stably at the final drug concentration to obtain drug-resistant cell lines. Detect the IC50 of drug-resistant cell lines and calculate the resistance index (RI), RI = IC50 of drug-resistant cell lines/IC50 of parental cell lines, and RI  > 5 is considered to be in accordance with drug-resistant strains.

### Animal models

All animal studies were approved by the Ethics Committee of Tianjin Medical University Cancer Institute and Hospital and were conducted by skilled experimenters under an approved protocol in accordance with the principles and procedures outlined in the NIH Guide for the Care and Use of Laboratory Animals.

For the subcutaneous xenograft animal model, four-week-old male BALB/C nude mice were maintained in a barrier facility on high-efficiency particulate air (HEPA)-air-filtered racks. The tumor cells were seeded into a single-cell suspension at a density of 1 × 10^7^ cells/mL. A total of 1 × 10^6^ cells were subcutaneously injected into each mouse to induce tumor development. After one week, GEM (50 mg/kg) was intraperitoneally administered to the treated group, and the same volume of saline was administered to the control group twice a week. Tumor size was measured weekly using calipers. The weights of the mice were recorded before and at the end of the experiment.

For the patient-derived xenograft (PDX) animal model, samples were repeatedly rinsed with pre-cooled sterile saline and immediately placed in a pre-cooled specialized preservative solution. The patient tumor tissues were placed individually into plates containing RPMI 1640 medium and transported to the laboratory at 4 °C. The tumor tissues were cleaned, and each one was cut into 3–5 mm tumor masses. Tumor masses were implanted at four subcutaneous points in each of the two female NSG mice. Tumor growth was monitored daily. When the tumors reached a threshold volume of 1 cm^3^, the tumor-bearing mice were sacrificed, and the tumors were dissected. Next, the tumors were removed, cut into small pieces (3 × 3 × 3 mm), and subcutaneously inoculated into five female NSG mice. The left and right flanks of each mouse were inoculated. When the tumor volume reached 300 mm^3^, GEM alone, Ro5-3335 (5 mg/kg) alone or GEM + Ro5-3335 was intraperitoneally administered to each treated group, and saline was administered to the control group twice a week. After three weeks, the tumors were harvested and weighed. The rate of tumor inhibition (ITR) of the drug was calculated by 100% × (average tumor weight of the control-average tumor weight of the treated)/ (average tumor weight of the control).

### Statistical analysis

Analyses were performed using SPSS software (version 21.0, SPSS Inc., Chicago, IL, USA) and GraphPad Prism 8.0. All data were obtained from at least three independent experiments. The results are shown as means ± SEM. Survival was analyzed using Kaplan–Meier and log-rank tests. Single-or multivariate analyses and the COX model were used to evaluate the hazard of variance related to survival. Student’s *t*-test or ANOVA variance was used for comparisons between groups. *p* < 0.05 was considered to indicate statistically significant differences.

## Results

### Aberrant RUNX1 expression indicates poor survival in PDAC

**Fig. 1:**
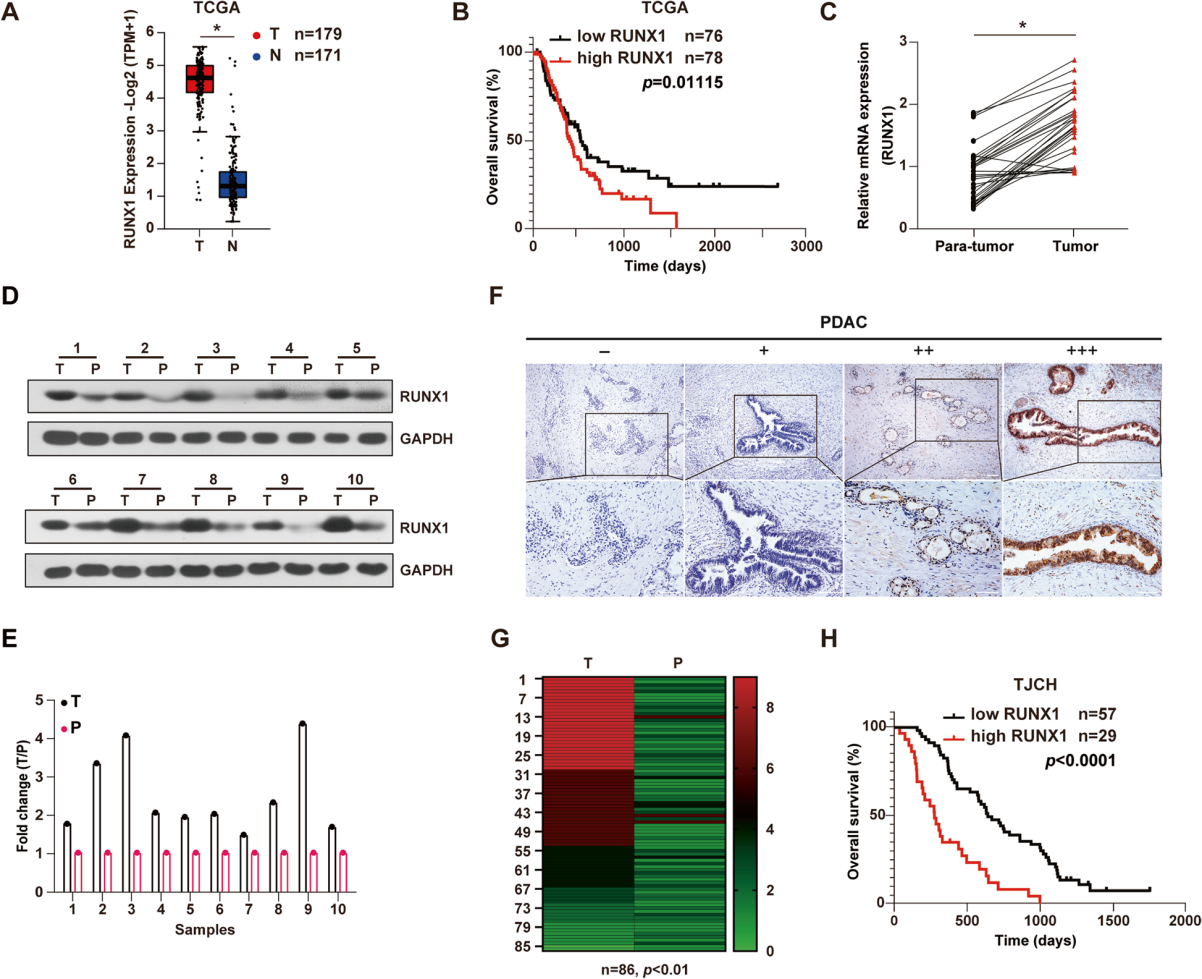
Aberrant RUNX1 expression in pancreatic adenocarcinoma correlates with disease progression. **A** mRNA expression analysis of RUNX1 in PDAC(T) tissues and normal pancreatic tissues(N) based on the TCGA dataset. **B** Survival analysis of PDAC patients with low and high RUNX1 expression based on the TCGA dataset. **C** mRNA expression of RUNX1 in PDAC tissues and matching para-tumor tissues by qRT-PCR assay. **D**-**E** RUNX1 expression in PDAC and matching para-tumor tissues by western blot (**D**), and the column diagram of RUNX1 expression generated by the grey value measured using Image J software (**E**). **F** Representative IHC images showing RUNX1 expression (-, + , +  + , +  + +) in PDAC tissues. Scale bar of the above, 100 µm; Scale bar of the below, 200 µm. **G** The comparative heatmap showing RUNX1 expression in 86 cases of PDAC tissues and matching para-tumor tissues ranging from green (low expression) to red (high expression). The column clustering generated by the IHC scores of the RUNX1 staining. **H** Survival analysis of PDAC patients with low and high RUNX1 expression based on the dataset of Tianjin Cancer Hospital (TJCH). Student's t-test were used in the column diagram; *, *p* < 0.05

The RUNX1 transcription factor has been found to behave depending on the type of disease [9]. Although RUNX1 is well-known in hematology, its versatility in solid cancers, including PDAC, remains unclear. To elucidate the role and mechanism of RUNX1 in PDAC, TCGA data were first analyzed in patients with PDAC. Observed expression of RUNX1 was higher in cancer tissues than normal tissues (Fig. 1A), which was linked with a significantly shorter survival (Fig. 1B). Consistent with this, qRT-PCR and western blot showed the aberrant high expression of RUNX1 in PDAC tissues at both mRNA and protein level (Fig. 1C-E). Given that RUNX1 and the other two isoforms comprise the runt-related transcription factor family, the expression of RUNX2 and RUNX3 in PDAC was also investigated. Among them, RUNX1 was relatively more highly expressed than the other two RUNX genes (Supplemental Fig. 1A), and the significant difference in RUNX1 expression between tumor and para-tumor tissues excluded the role of RUNX2 and RUNX3 (Supplemental Fig. 1B-C), suggesting the importance of abundant RUNX1.

To explore the clinical roles of RUNX1 in PDAC, 86 samples from patients with PDAC were collected and immunohistochemistry (IHC) was performed. The staining of RUNX1 was scored as described previous study [16]. IHC assays showed that RUNX1 was predominantly located in the cell nucleus and partly in the cytoplasm (Supplemental Fig. 1D). RUNX1 was mainly expressed in cancerous tissues and some mesenchymal cells, and rarely in normal pancreatic tissues. In general, RUNX1 expressed higher in tumor tissues compared to the para-tumor tissues (Fig. 1F-G). However, RUNX1 was not widely expressed in PDAC, with intense staining in 33.7% of cases (Table 1). RUNX1 expression positively correlated with histologic grade (*r* = 0.388), tumor size (*r* = 0.319), and lymph node metastasis (*r* = 0.304). Increased intensity of RUNX1 staining was observed in the higher degree of malignancy groups, suggesting the pro-malignant features of RUNX1 function. Subsequently, univariate and multivariate analyses were conducted using the Cox proportional hazards model, which demonstrated that high RUNX1 expression was a significant independent risk factor in the prognosis of PDAC patients (*p* < 0.01) (Table 2). The effect of RUNX1 on overall survival (OS) of patients with PDAC was then evaluated. Negative and weak staining in the low RUNX1 expression group were identified, and, according to the scores, the median and strong staining comprised the high RUNX1 expression group. Survival analysis revealed that higher RUNX1 expression predicted shorter survival, which was consistent with the TCGA data (Fig. 1H). Therefore, RUNX1 is closely related to the malignant clinical features of PDAC, and its abundance predicts poor survival.

**Table 1:** The correlations between RUNX1 expression and clinicopathological features of patients with PDAC

	RUNX1		***p***	*r*
Parameters	-/ +	+ + / + + +		
Age, years			0.949	-0.007
< 60	33	17		
≥ 60	24	12		
Gender			0.956	-0.006
Male	37	19		
Female	20	10		
Histologic grade			0.000	0.388
G1-2	39	8		
G3-4	18	21		
Tumor size			0.010	0.319
T1-2	55	22		
T3	2	7		
LN metastases			0.005	0.304
N0	36	9		
N1	21	20

**Table 2:** The univariate and multivariate analysis of prognostic factors in the patients with PDAC

	OS	
Univariate analysis	HR (95.0%CI)	***p*** value
Age (≥ 60)	0.954(0.603–1.510)	0.842
Gender(male)	0.754(0.475–1.198)	0.232
Histologic grade(G3-4)	0.603(0.384–0.948)	0.029
Tumor size(> 3.5 cm)	0.157(0.074–0.335)	0.000
LN metastases(N1)	0.503(0.320–0.790)	0.003
RUNX1 expression(high)	0.289(0.175–0.479)	0.000
Multivariate analysis		
RUNX1 expression(high)	0.302(0.171–0.534)	0.000
Histologic grade(G3-4)	1.060(0.631–1.780)	0.826
Tumor size(> 3.5 cm)	0.203(0.089–0.466)	0.000
LN metastases(N1)	0.677(0.412–1.113)	0.124

### RUNX1 in vitro facilitates the GEM resistance in PDAC

**Fig. 2:**
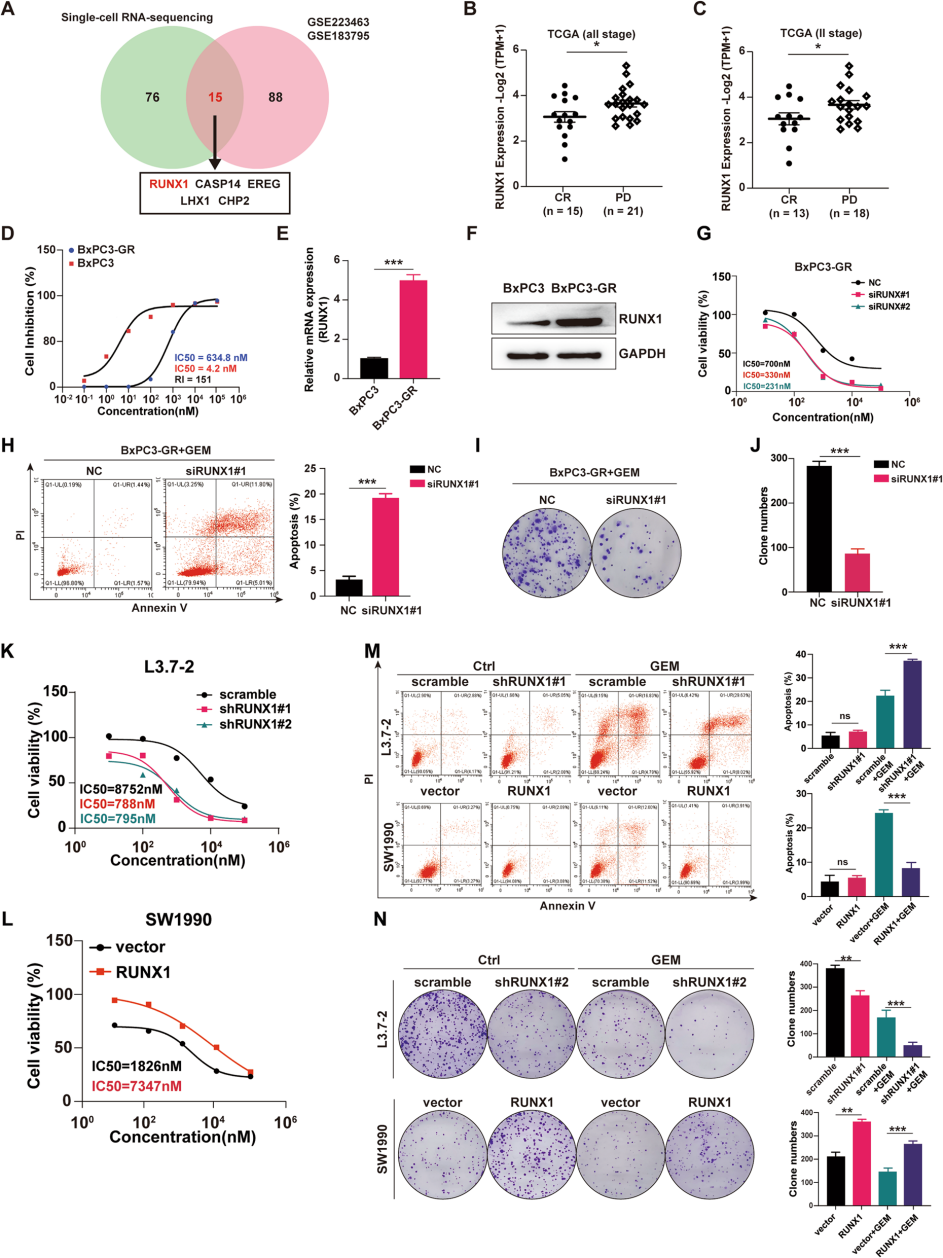
RUNX1 in vitro facilitates the gemcitabine resistance in PDAC. **A** Venn diagram showing the top 5 gemcitabine-resistance related genes. The diagram was generated by the overlap of differential expression genes from scRNA-seq data (HRA000433) and GEO datasets (GSE223463, GSE183795). **B**-**C** mRNA expression of RUNX1 in gemcitabine-treated all-stage (**B**) and Stage II (**C**) PDAC patients with complete relief (CR) or clinical progressive disease (PD). **D** IC50 value of gemcitabine in BxPC3 and BxPC3-GR cell lines by the cell counting kit-8 assay. **E** The mRNA expression of RUNX1 in BxPC3 and BxPC3-GR cell lines as determined by qRT-PCR. **F** Immunoblot of RUNX1 in BxPC3 and BxPC3-GR cell lines. **G** IC50 value of gemcitabine in BxPC3-GR cells transfected with siRNA targeting RUNX1 (siRUNX1#1, siRUNX1#2) by the cell counting kit-8 assay. **H** Apoptosis of BxPC3-GR cells transfected with siRUNX1#1 was assessed by flow cytometry after gemcitabine treatment. The column diagram represents the average cell apoptosis rates of BxPC3-GR cells transfected with siRUNX1 under gemcitabine treatment compared with the control (NC). **I**-**J** Clonogenic assay of BxPC3-GR cells transfected with siRUNX1#1, seeded at 1000cells/ well, then treated with gemcitabine (200 nM). Colonies were stained with crystal violet (0.5%) after 14 days and counted using ImageJ software. **K**-**L** Cell viability of L3.7–2-shRUNX1 (#1,#2) cell line or SW1990-RUNX1 cells treated with different concentration of gemcitabine for 72 h, compared with the control (scramble or vector). IC50 values were calculated and shown in the Figure. **M** Cell apoptosis of L3.7–2 cells with shRUNX1#1 or SW1990 cells with RUNX1 overexpression (SW1990-RUNX1) by flowcytometry, under gemcitabine treatment (2 µM, 48 h). The average cell apoptosis rate of each group was shown in the column diagram. **N** Clonogenic assay of L3.7–2-shRUNX1#1 or SW1990-RUNX1 cells seeded at 1000 cells/well, under gemcitabine treatment (200 nM). Colonies were stained with crystal violet (0.5%) after 14 days and counted using the ImageJ software. Student’s t-test was used in the column diagram; *, *p* < 0.05; **, *p* < 0.01; ***, *p* < 0.001; ns, no significance

In PDAC, several studies have shown that RUNX1 plays an oncogenic role in tumor growth and metastasis [14, 17, 18]. Given that GEM-resistance is always followed by clinical disease progression in PDAC, it is well worth investigating whether RUNX1 is involved in the development of GEM-resistance. We got 14 cases of scRNA-seq data for PDAC and the follow-up information of the corresponding patients, the data was divided into two groups: response group (including stable disease and reduced tumor) and non-response group (disease progression), according to the patients’ response to GEM-based chemotherapy. We thus obtained a cluster of differentially expressed genes (DEGs) related with drug sensitivity. Meanwhile, two public RNA-seq datasets (GSE223463, GSE183795) were screened for another cluster of treatment-related DEGs. These two clusters were overlapped to generate a small group of genes in which RUNX1 ranked first (Fig. 2A). The details of PDAC patients from TCGA data were analyzed and RUNX1 expression of tumor tissues from PDAC patients grouped by response to GEM was compared. As expected, it was found that the expression of RUNX1 in GEM-treated patients with complete relief (CR) was significantly different from that in patients with clinical progressive disease (PD). There was higher expression of RUNX1 in the PD group than in the CR group in patients of all stages, as well as in stage II patients (Fig. 2B-C). Hence, all the above unbiased analysis suggests that RUNX1 is the most critical GEM-resistance related gene.

Next, we investigated whether RUNX1 expression imparts resistance to GEM in PDAC cells. To achieve this, a GEM-resistant (GR) cell model was established from the human pancreatic cancer cell line BxPC3 using the dose increment method. The resistance index was 151 (IC50 _BxPC3-GR_/IC50 _BxPC3_) (Fig. 2D), suggesting that the GEM-resistant cell model BxPC3-GR succeeded. Of note, it was found that mRNA level and protein abundance of RUNX1 in BxPC3-GR cells were higher than in BxPC3 cells (Fig. 2E-F). After RUNX1 expression was reduced by siRNA, the IC50 value of GEM in BxPC3-GR cells decreased by 50% compared to that of the control (Fig. 2G). Furthermore, we observed an increase in apoptosis rates and a reduction in colony counts in BxPC3-GR cells transfected with siRNA targeting RUNX1 after GEM treatment (Fig. 2H-J, Supplemental Fig. 2A-B). In contrast, after forced expression of RUNX1, the parental cell line BxPC3 showed increase of cell proliferation and reduced cell death by GEM (Supplemental Fig. 2C-E).

Also established was silencing construct targeting RUNX1 and its stable integration into L3.7–2 and CFPAC1 cell lines, and an overexpression construct into SW1990 and MIA-PaCa2 cell lines. The cell viability assay showed a decrease in the IC50 value of GEM in the shRUNX1 group but an obvious increase in the RUNX1 overexpression (RUNX1-OE) group (Fig. 2K-L, Supplemental Fig. 2F-G). The average cell apoptosis rate increased to 35–38% in the L3.7–2-shRUNX1 group, but decreased to 7–9% in the SW1990-RUNX1 group treated with GEM (Fig. 2M). Similar results were obtained for CFPAC1-shRUNX1 and MIA-PaCa2-RUNX1 cells (Supplemental Fig. 2H). In particular, no difference in the apoptosis rate was observed without GEM treatment when RUNX1 was overexpressed or decreased. These results suggested a specific role for RUNX1 in GEM-induced apoptosis. Colony formation assays were performed using cell lines transfected with shRUNX1 or RUNX1-OE. Consistent with the BxPC3-GR cells, a reduction in colony counts was found in the shRUNX1 cells treated with gemcitabine, whereas the opposite tendency was observed in RUNX1-OE cells (Fig. 2N, Supplemental Fig. 2I). Therefore, RUNX1 could weaken the effects of gemcitabine on PDAC cells, conferring a survival advantage to cells following GEM treatment, which is indispensable for the development of GEM resistance.

### RUNX1 impedes the gemcitabine response to PDAC in vivo

**Fig. 3:**
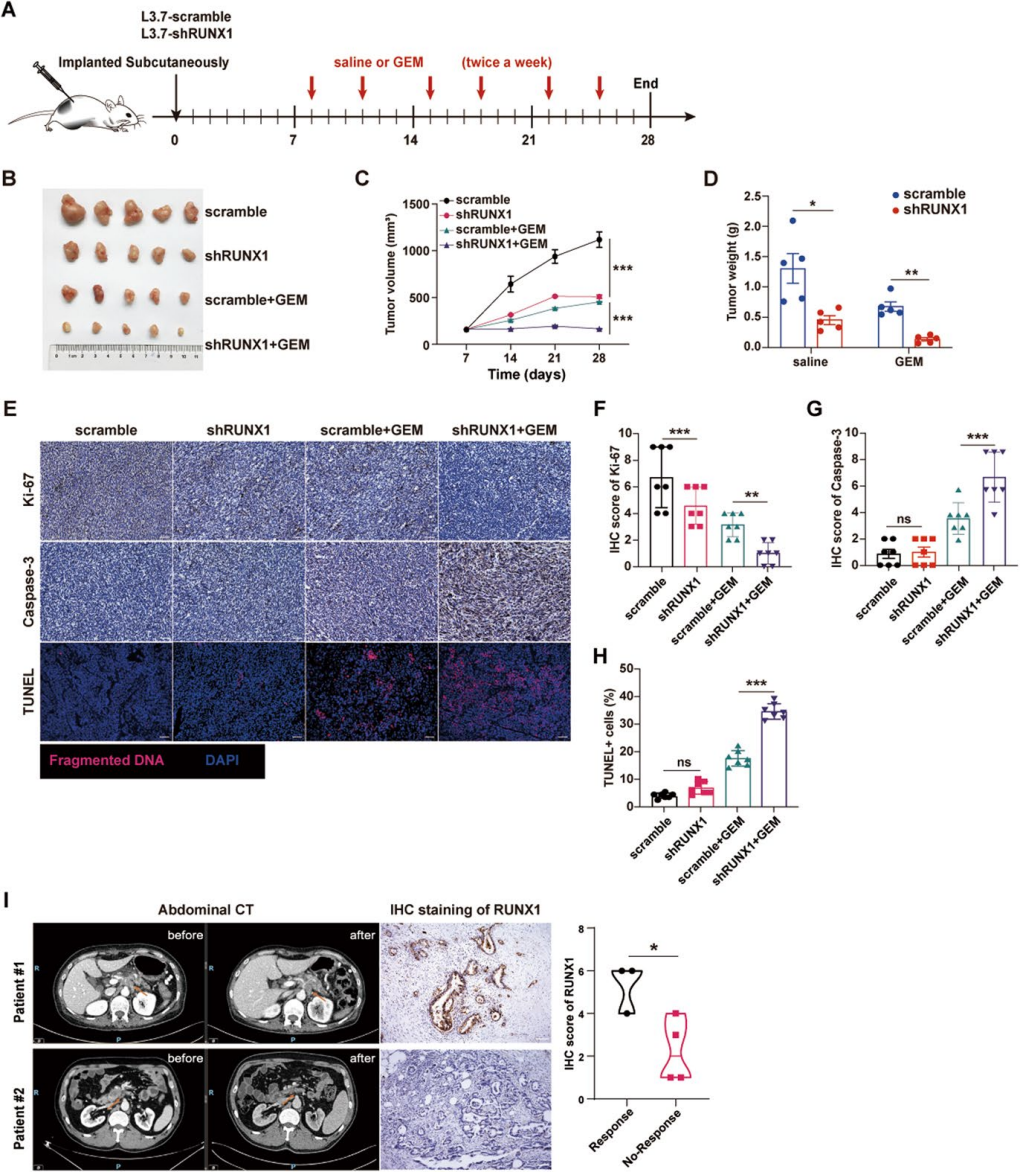
RUNX1 impedes the gemcitabine response to PDAC in vivo. **A** Diagram of a mouse xenograft treated with gemcitabine. **B**-**C** Images of the tumor tissues and the tumor growth curve of the shRUNX1 group compared to the control (scrambled) under gemcitabine treatment. **D** Comparison of tumor weights of the shRUNX1 group compared to the control (scramble), with or without gemcitabine treatment. **E** Representative images of Ki-67, Caspase3 staining and TUNEL staining of the tumor tissues of the scrambled and shRUNX1 groups with or without gemcitabine treatment. **F**–**H** Analysis of Ki-67, Caspase3 staining, and the average number of apoptotic cells in the scrambled and shRUNX1 groups, with or without gemcitabine treatment, are displayed. **I** Representative images of enhanced abdominal computed tomography (CT) scans of PDAC patients (#1, #2) before and after two cycles of gemcitabine-based chemotherapy. The orange arrow indicates the lesion. Representative IHC images of RUNX1 staining in patients #1 and #2 are shown on the right. The violin plots showing the RUNX1 expression in response group (including stable or decreased tumor) and no-response group (progressed disease). Student’s t-test was used in the column diagram; scale bar, 100 µm. *, *p* < 0.05; **, *p* < 0.01; ***, *p* < 0.001; ns, no significance

Based on the in vitro findings, we sought to confirm the effect of RUNX1 on gemcitabine response in vivo. L3.7–2 shRUNX1 cells were subcutaneously implanted into nude mice. After tumor formation, the mice were treated with gemcitabine or saline for the indicated times (Fig. 3A). It was found that the tumor grew slowly, and the tumor weight was lighter in the shRUNX1 group without gemcitabine treatment, and the difference was more significant with gemcitabine treatment (Fig. 3B-D). In addition, the inhibition tumor rate (ITR) of gemcitabine was calculated (shRUNX1 group, 78.4% *vs* control group, 64.15%), which showed a better response to gemcitabine in the shRUNX1 group. Furthermore, Ki-67 and Caspase3 activity, representing cell proliferation and apoptosis, respectively, were measured and scored according to the intensity and area. Lower scores for Ki-67 and higher scores for Caspase3 activity in shRNA cells treated with GEM were observed. TUNEL assays also revealed increased apoptosis in cells in the shRUNX1 group treated with gemcitabine (Fig. 3E-H). Additionally, IHC staining was performed for RUNX1 in PDAC tissues from patients with different responses to neoadjuvant chemotherapy. There were seven patients enrolled in this analysis. These patients were primarily diagnosed with PDAC by biopsy and underwent tumor resection after two cycles of gemcitabine-based chemotherapy. They were grouped by the response to therapy: response group (including stable disease and reduced tumor) and no response (progressed disease) group. The alignment of the response assessment and RUNX1 staining showed that patients with disease progression exhibited strong staining for RUNX1 in tumor tissues; however, those with response to chemotherapy showed weak staining (Fig. 3I). Overall, these data revealed increased chemoresistance related with RUNX1 abundance. Tumor with low RUNX1 expression might respond better to gemcitabine therapy, suggesting the potential value of RUNX1 in PDAC.

### RUNX1 imparts gemcitabine resistance in PDAC through ER stress

**Fig. 4:**
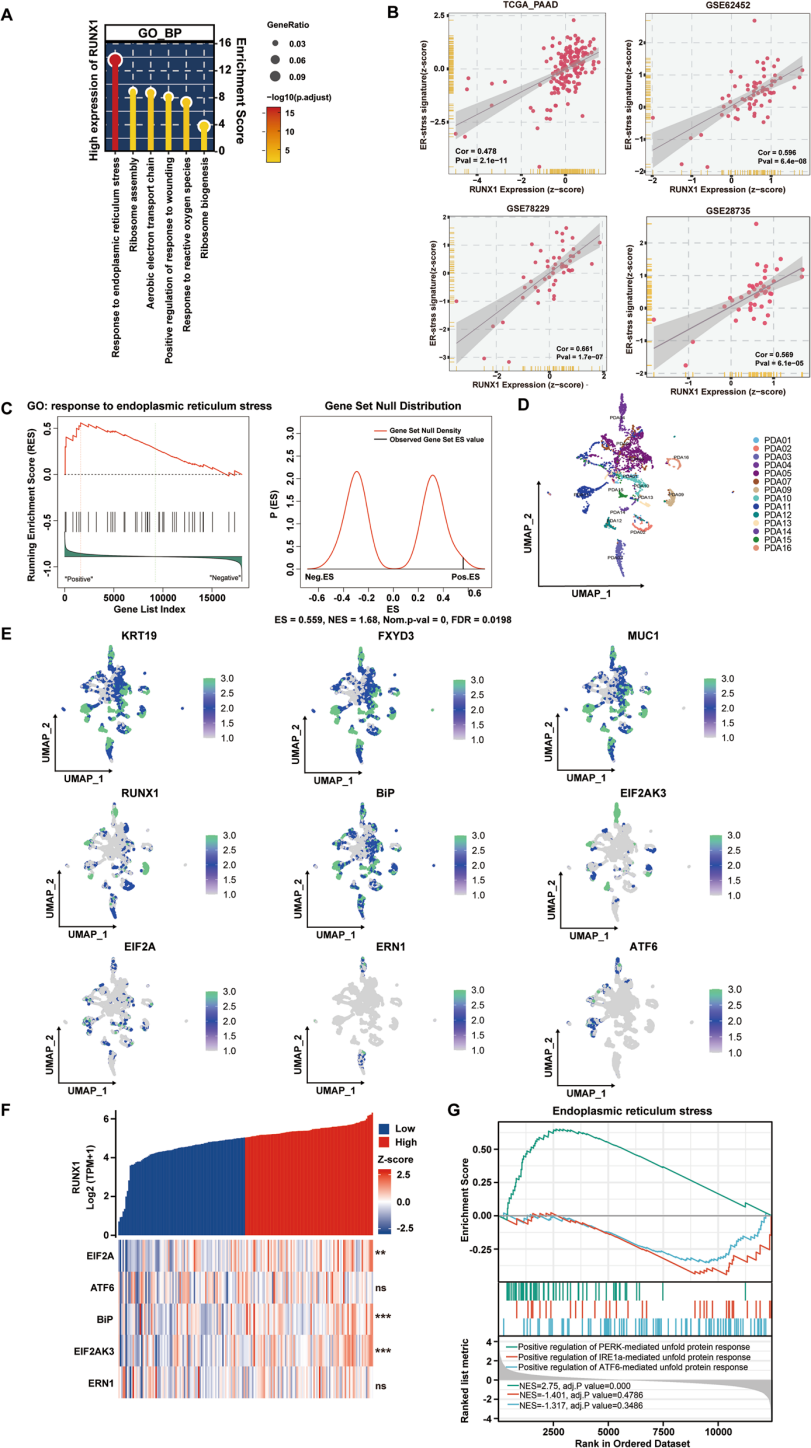
RUNX1 is associated with ER stress pathway in PDAC at the single-cell level. **A** GO Enrichment analysis (biological process) of RUNX1 based on TCGA dataset. The color ranges from red (strong significance) to yellow (weak significance). **B** Correlation analysis of RUNX1 and ER stress signatures based on public datasets (TCGA, GSE62452, GSE78229, GSE28735). **C** Differential gene expression analysis between high and low RUNX1-expressing malignant ductal epithelial cells. Gene Set Enrichment Analysis (GSEA) was performed with adjusted p value < 0.05 and FDR < 0.25 considered significant. **D** Single-cell RNA sequencing (scRNA-seq) analysis of 14 PDAC samples. The cells were sorted by EPCAM^+^ and clustered using the R software Seurat, with patient ID as the marker. **E** Feature plot analysis of KRT19, FXYD3, and MUC1 expression in ductal and malignant epithelial cells isolated from 14 PDAC tissue samples. Higher expression levels are indicated by brighter green shading. RUNX1 and the core molecules of the ER stress pathway, including BiP, EIF2ΑK3(PERK), EIF2Α(eIF2α), ERN1(IRE1α), and ATF6 were identified and visualized based on their expression levels. **F** Correlation between RUNX1 and ER stress pathway-related genes, including BiP, EIF2ΑK3, EIF2Α, ERN1, and ATF6, was analyzed using bulk RNA-seq data from 171 patients with PDAC obtained from TCGA. Co-expression heat maps were generated using the R software package heatmap, with red indicating high expression and blue indicating low expression. **G** Correlations between RUNX1 and the three UPR pathways were further analyzed. Correlation gene set enrichment analysis (GSEA) plots were generated and visualized using the R software package, GSEA

The RUNX1 transcription factor is involved in cellular processes through different signaling pathways; however, the mechanism of RUNX1 in GEM resistance of PDAC remains to be elucidated. To investigate the pathway that is dependent on RUNX1 function, the Gene Ontology (GO) enrichment analysis based on the TCGA RNA-seq data for PDAC was performed and revealed the first rank of ER stress signaling among the pathways of significance (Fig. 4A). Meanwhile, the data from TCGA, GSE62452, GSE78229 and GSE28735 all supported a strong correlation of RUNX1 with ER stress signatures (Fig. 4B). Also, 14 cases of scRNA-seq data for PDAC was performed after Epcam^+^ cell sorting. Based on differential gene expression analysis of malignant ductal epithelial cells with high and low RUNX1 expression, the ER stress signaling was identified as a key enriched pathway in cells with RUNX1 highly expressed by GSEA (Fig. 4C). ER stress is a cytoprotective pathway initiated by various stimuli that confers drug resistance in various cancers, including PDAC. Generally, under ER stress, BiP binds to the mis-unfolded protein and releases three downstream UPR-sensors (PERK, IRE1α, and ATF6) which then activate the three UPR pathways: PERK branch, IRE1α branch and ATF6 branch; however, the predominance of the activated pathway depended on cell-context and the external environment [19]. Using Seurat software, dimensionality reduction clustering was performed and the clusters labeled according to patient ID (Fig. 4D). Using KRT19 as a marker for ductal epithelial cells, and FXYD3 and MUC1 as markers for malignant ductal epithelial cells, their expression levels were displayed by using a feature plot function. Also highlighted were the core molecules of the ER stress related genes: HSPA5(BiP), EIF2ΑK3(PERK), EIF2Α(eIF2α), ERN1(IRE1α), and ATF6. We found RUNX1 was well overlapped with BiP, PERK and eIF2α in the tumor cells relatively to other ER stress related markers (Fig. 4E). Additionally, significant difference of BiP, PERK and eIF2α was showed between RUNX1-high and -low expression group, whereas IRE1α and ATF6 were not (Fig. 4F). It was inferred that RUNX1 more strongly correlated with PERK/eIF2α signaling, compared to the IRE1α or ATF6 mediated branch, which was also been supported by the further GSEA (Fig. 4G). The same bioinformatics analysis was performed on the scRNA-seq data from CRA001160 and revealed a tight association between RUNX1 and ER stress (Supplemental Fig. 3A-C). Also, the PERK mediated signaling was highlighted in this analysis (Supplemental Fig. 3D). Therefore, it is supposed that the RUNX1 functioned through the PERK/eIF2α branch.

**Fig. 5:**
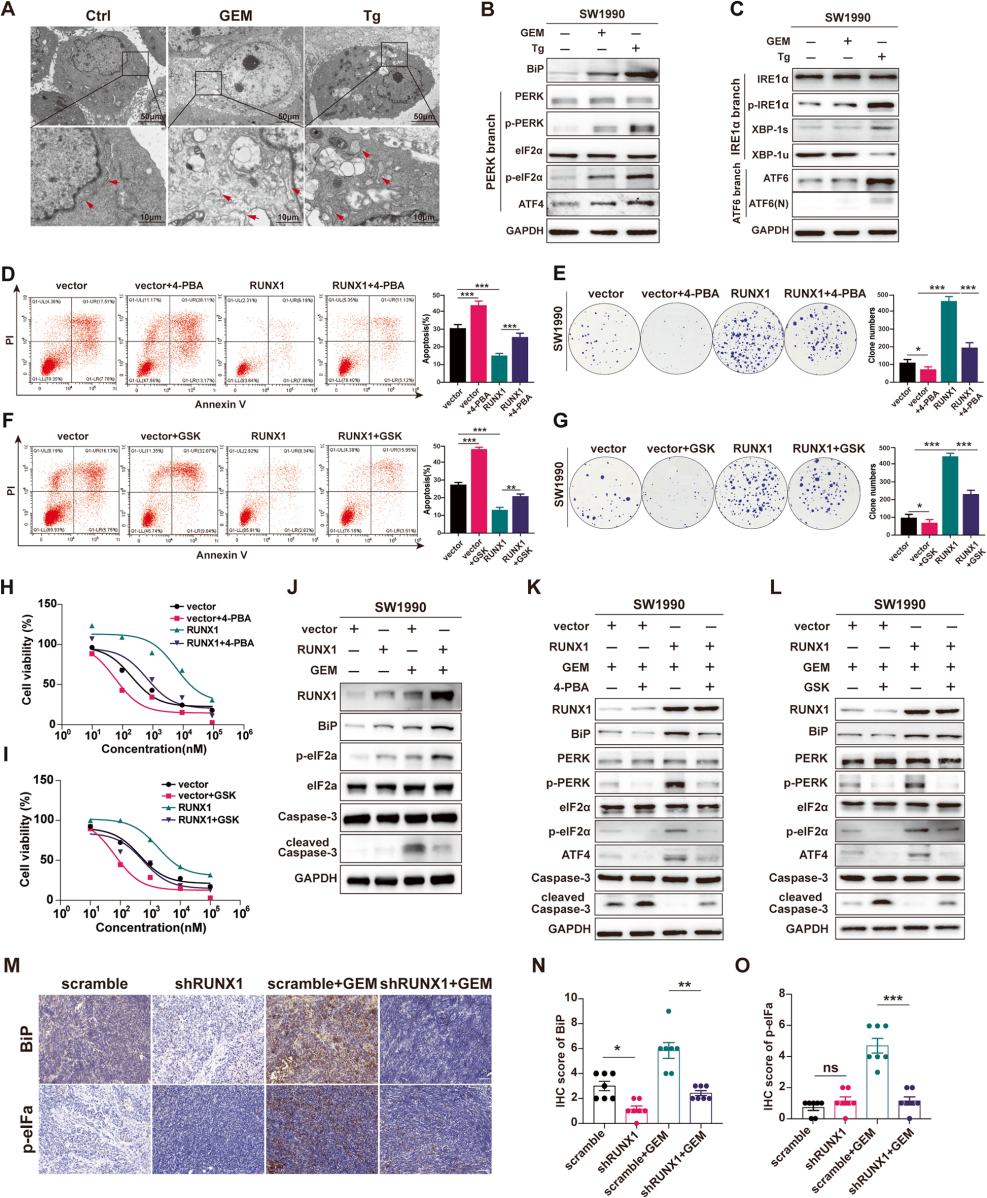
RUNX1 imparts gemcitabine resistance in PDAC through ER stress. **A** Representative morphological images of the ER structure using transmission electron microscopy. SW1990 cells were treated with 2 µM gemcitabine for 48 h, or with 300 nM thapsigargin (Tg) for 6 h as the positive control, or normal media for 12 h as the negative control. The red arrow indicates ER structure. Scale bar of the above is 50 µm, scale bar of the below is 10 µm. **B**-**C** The immunoblot analysis of BiP expression and three UPR branches: PERK branch, IRE1α branch and ATF6 branch on SW1990 cell lines treated with gemcitabine (2 µM, 24 h), Tg(100 nM, 6 h) and normal media. **D** and **F** The cell apoptosis of SW1990-RUNX1 cell lines treated with combination of gemcitabine (2 µM) and 10 nM 4-PBA (**D**) or 10 µM GSK2606414 (**F**) for 48 h. The average cell apoptosis rate in each group is shown in the right column. **E** and **G** Clonogenic assay of SW1990-RUNX1 cells, which were seeded at 1000 cells/well, in gemcitabine (200 nM), and then 4-PBA (**E**) or GSK2606414 (**G**) was added. Colonies were stained with crystal violet (0.5%) after 14 d and counted using ImageJ software. **H**-**I **Cell viability of SW1990-RUNX1 cells treated with a combination of gemcitabine and 10 nM 4-PBA (**H**) or 10 µM GSK2606414 (**I**) for 72 h. JThe immunoblot analysis of ER stress related markers (BiP, p-eIF2α) and cell apoptosis marker (cleaved Caspase 3) on SW1990-RUNX1 cell line treated with gemcitabine (2 µM, 48 h).**K**-**L** The immunoblot analysis of the BiP/PERK/eIF2α axis and cell apoptosis marker(cleaved Caspase 3) on SW1990-RUNX1 cell line treated with combination of gemcitabine and 10 nM 4-PBA (**K**) or 10 µM GSK2606414 (L) for 48 h. **M**–**O** Representative images of BiP and p-eIF2α staining in subcutaneous xenograft tissues of the scramble and shRUNX1 groups with or without gemcitabine (M, tissues are shown in Fig. 3). The IHC scores for BiP (**N**) and p-eIF2α (**O**) staining in each group were evaluated and are shown in the right column of the diagram. Student’s t-test were used in the column diagram; *, *p* < 0.05; **, *p* < 0.01; ***, *p* < 0.001; ns, no significance. GSK, GSK2606414

To validate these findings from the bioinformatics analysis, a series of experiments were performed. First, morphological changes were observed in ER stress induced by gemcitabine, including ER enlargement, deformation, and vesicle formation, using transmission electron microscopy (TEM) for SW1990 cell line (Fig. 5A) and L3.7–2 cell line (Supplemental Fig. 4A). Besides that, increase of BiP expression and activation of three UPR branches including PERK branch, IRE1 α branch and ATF6 branch were used to confirmed the ER stress status. The immunoblot revealed changes of BiP and three branches in the cells treated with Tg (the positive control); however, only obvious changes of BiP and PERK signaling were detected in the GEM treated cells (Fig. 5B-C, Supplemental Fig. 4B). This was consistent with the upregulation of the eIF2 pathway in pancreatic cancer cells treated with gemcitabine in other studies [20].

To confirm the role of ER stress as a likely mechanism for RUNX1 imparting GEM resistance, we then evaluated whether chemically targeting ER stress could reverse the response of pancreatic cancer cells to GEM. The ER stress inhibitor 4-PBA [21] had no effect on PDAC cells at a set of different concentrations (Supplemental Fig. 4C-D). Cell apoptosis assays showed an increase in the number of apoptotic RUNX1-OE cells treated with 4-PBA and gemcitabine, compared to those treated with gemcitabine alone (Fig. 5D, Supplemental Fig. 4E). Clonogenic assays using RUNX1-OE cells were performed to determine whether the combination of 4-PBA and gemcitabine displayed enhanced antiproliferative effects. An obvious reduction in the colony counts of RUNX1-OE cells were found under combination drug treatment (Fig. 5E, Supplemental Fig. 4G). Moreover, the cell viability assays of RUNX1-OE cells treated with 4-PBA and gemcitabine simultaneously demonstrated a decrease in cell survival compared to cells exposed to gemcitabine alone (Fig. 5H, Supplemental Fig. 4I).

Furthermore, the dominance of the PERK/eIF2α pathway in RUNX1 mediated gemcitabine resistance was confirmed using the PERK inhibitor GSK2606414, as well as the results reported in previous study [22]. Increased apoptosis rates were observed in cells treated with gemcitabine and GSK2606414 compared to those in cells treated with gemcitabine alone (Fig. 5F Supplemental Fig. 4F). Clonogenic assays showed a significant reduction in colony counts when the cells were exposed to gemcitabine and GSK2606414 (Fig. 5G, Supplemental Fig. 4H). In addition, cell viability assays showed decreased growth rates when cells were treated with gemcitabine and GSK2606414, compared to cells exposed to gemcitabine alone (Fig. 5I, Supplemental Fig. 4J).

Also detected were the change of the PERK/eIF2α signaling in shRUNX1 or RUNX1-OE cells with gemcitabine alone, and combination of gemcitabine and 4-PBA or GSK2606414. It was observed that the protein expression of BiP and p-eIF2α rose in RUNX1-OE cells and lowered in shRUNX1 cells. Investigation of caspase 3 activity showed an increase in cleaved Caspase 3 protein levels in shRUNX1 cells, but a decrease in RUNX1-OE cells when cells were treated with gemcitabine (Fig. 5J, Supplemental Fig. 4K). Also found was that 4-PBA attenuated the whole BiP/PERK/ eIF2α signaling which was consistent with previous reports [23, 24], and GSK2606414 inhibited PREK phosphorylation, which subsequently weakened eIF2α phosphorylation, a core effector in the PERK-mediated pathway as well as the upstream effector of activating transcription factor 4 (ATF4). Finally, the activity of Caspase 3 increased with both combination treatments (Fig. 5K-L).

Eventually, BiP/PERK/ eIF2α signaling was detected in vivo, IHC staining of BiP and p-eIF2α were performed in the tumor tissues from the xenograft models with L3.7–2-shRUNX1 cells (Fig. 5M). Lower IHC scores were found for BiP in the shRUNX1 group than in the control group, regardless of gemcitabine treatment (Fig. 5N). While p-eIF2α staining was weaker in the shRUNX1 group treated with gemcitabine than in the control group, no significant difference was observed without gemcitabine treatment (Fig. 5O), which further highlighted the importance of PERK/ eIF2α signaling under stress.

Altogether, these results indicate that RUNX1 modulates ER stress via the PERK/eIF2α axis, driving adaptive capacity of tumor cells and promoting acquisition of GEM resistance in PDAC.

### RUNX1 transcriptional regulates the expression of BiP in the PDAC cells

**Fig. 6:**
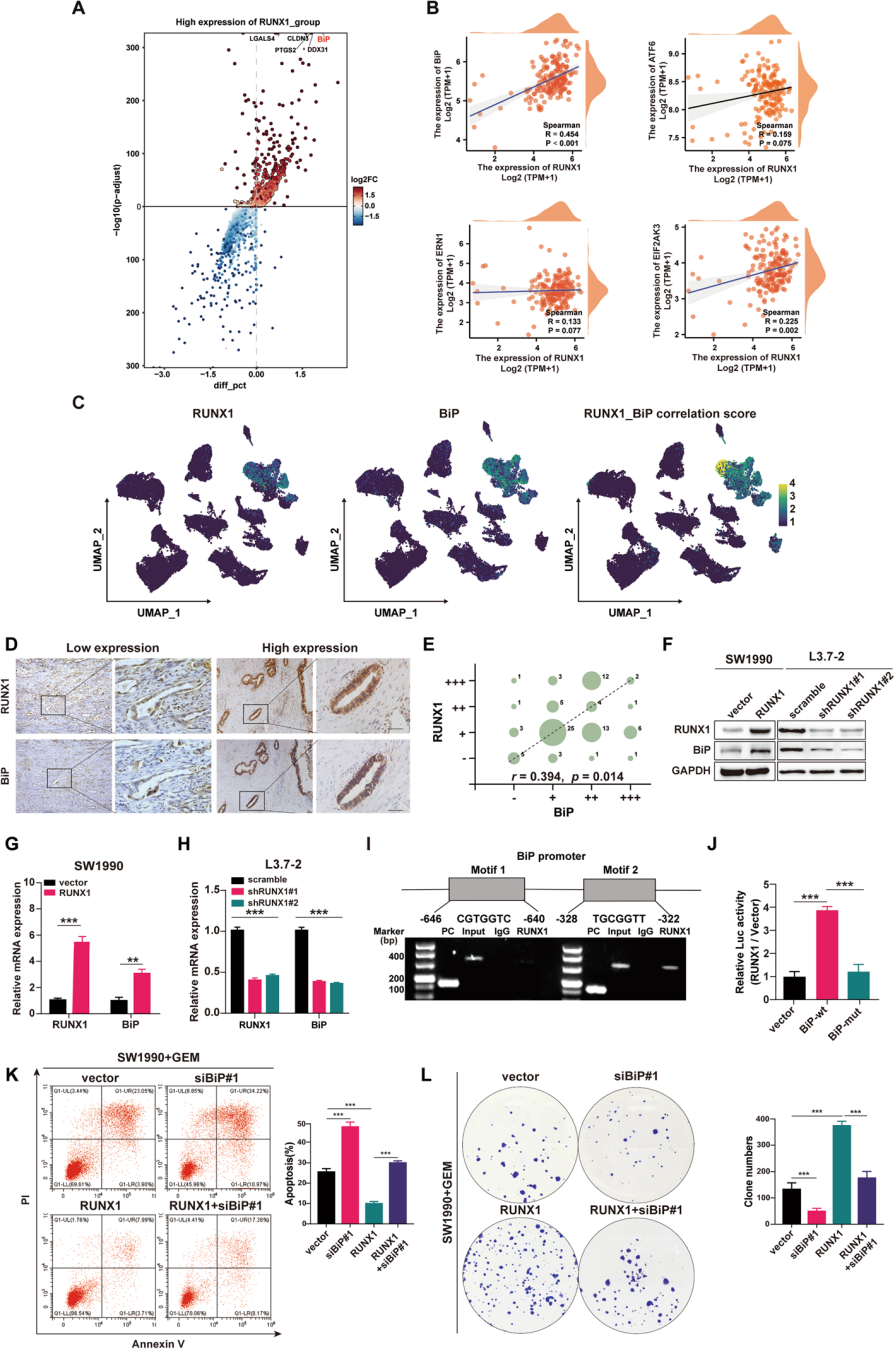
RUNX1 positively transcriptional regulates the expression of BiP in PDAC cells. **A** Volcano diagram showing the differential genes by RUNX1 alteration based on TCGA dataset. **B** Spearman correlation analysis of RUNX1 and ER stress related markers: BiP, EIF2ΑK3(PERK), ERN1(IRE1α), and ATF6), based on the TCGA dataset. **C** Feature plot showing the co-expression relationship of RUNX1 and BiP expression in malignant ductal epithelial cells in the downloaded single-cell dataset (CRA001160), color ranges blue (low) to yellow (high)represents correlation score. **D**-**E** Representative images of BiP and RUNX1 staining (low and high) on successive slides of human PDAC tissues (**D**). The bubble diagram (**E**) showing expression of RUNX1 and BiP in each sample. The bubble size represents the number of cases. **F** Immunoblot analysis of RUNX1 and BiP expression in SW1990-RUNX1 and L3.7-shRUNX1 cells. **G**-**H** mRNA levels of RUNX1 and BiP in SW1990-RUNX1 and L3.7-shRUNX1cell lines by RT-qPCR. **I** ChIP analysis of SW1990-RUNX1 cells. Chromatin was immunoprecipitated using an anti-RUNX1 antibody and subjected to PCR. **J** SW1990-RUNX1 cells were transfected with a pGL3-BiP-widetype (wt), pGL3-BiP-mutation or pGL3-control vector. The results are presented as a fold-change Firefly activity relative to cells transfected with the control vector after normalization to Renilla activity. **K** The cell apoptosis of SW1990-RUNX1 cells transfected with siRNA targeting BiP(siBiP#1) by flowcytometry, under gemcitabine treatment (2 µM, 48 h). The average apoptosis rate in each group is shown in the column diagram. **J** Clonogenic assay of SW1990-RUNX1 cells and SW1990-vector cells transfected with siBiP#1, under gemcitabine treatment (200 nM). Colonies were stained with crystal violet (0.5%) after 14 d and counted using ImageJ software. Student’s t-test was used in the column diagram; scale bar, 200 µm; *, *p* < 0.05; **, *p* < 0.01; ***, *p* < 0.001

Though the relationship between RUNX1 and ER stress has been discussed in previous report [13], the modulation mechanism has rarely been reported. To gain insight into the mechanism behind the RUNX1 modulation of ER stress, the differential expression genes analysis was conducted using the TCGA dataset for PDAC. As shown in the volcano diagram, among the dysregulated genes, obvious significant fold change of BiP was found in the cluster of upregulated genes by RUNX1 alteration (Fig. 6A). Moreover, a strong positive correlation was also found between RUNX1 and BiP (*r* = 0.454) relatively to other UPR activators (Fig. 6B). Additionally, there was a strong co-expression relationship of RUNX1 and BiP in malignant ductal epithelial cells in the download single-cell dataset for PDAC, as well as the result of our own scRNA-seq data (Fig. 6C, Supplemental Fig. 5A). We furtherly validated this relation in the PDAC tissues, and the IHC staining of RUNX1 and BiP were analyzed. Intensive RUNX1 staining were found with high IHC scores for BiP staining. Conversely, low IHC scores for BiP staining were associated with weak RUNX1 staining. Correlation analysis of IHC staining revealed a significant positive correlation between RUNX1 and BiP expression (*r* = 0.394, *p* = 0.014) (Fig. 6D-E). Furthermore, it was found that BiP expression was upregulated in RUNX1-OE PDAC cell lines at both the mRNA and protein levels, and vice versa (Fig. 6F-H). Collectively, these results supported RUNX1 positively regulation of BiP expression, indicating BiP might be a critical downstream target of RUNX1 during modulation of ER stress.

As known, BiP is a primary sensor of ER stress. It is reported that BiP is not only ER-located chaperone, but also has transcriptional function on PERK signaling [25]. Hence, we supposed that RUNX1 modulated the PERK/eIF2α signaling through BiP. However, RUNX1 regulation of BiP expression was unclear. RUNX1 is a co-transcription factor that contains a DNA-binding domain. We searched online for two possible RUNX1-binding sites in the BiP promoter area (http://jaspar.genereg.net/). ChIP-PCR was performed to detect a signal at one site (Fig. 6I), suggesting that RUNX1 could bind to the region of BiP promoter. Next, a dual luciferase assay in RUNX1-OE cells demonstrated higher Firefly/Renilla luciferase expression in cells transfected with pGL3-BiP-wt plasmids, whereas lower expression was observed in cells transfected with pGL3-BiP-mut plasmids (Fig. 6J).

To confirm the effect of BiP on GEM-resistance from RUNX1, the expression of BiP was knocked down by small interfering RNA (siRNA) in RUNX1-OE cells, and then cell apoptosis assays were carried on under gemcitabine treatment. The results showed that reduced apoptosis rates in RUNX1-OE cells were reversed by a decrease in BiP expression (Fig. 6K, Supplemental Fig. 5B-C). In addition, colony formation analysis of RUNX1-OE cells transfected with siRNA targeting BiP was performed using RUNX1-OE cells transfected with scrambled siRNA as a control. Decreased colony counts in RUNX1-OE cells with BiP knockdown compared to control cells were observed (Fig. 6L, Supplemental Fig. 5D-E). As well as in the cell apoptosis assay, cell viability test revealed the increase cell viability in RUNX1-OE cells were reversed by BiP knockdown. What’s more, we found interference with BiP resulted in decrease of cell viability and cell proliferation, and increased cell apoptosis rates (Supplemental Fig. 5F-G).

Taken together, our finding demonstrates that RUNX1 is a transcriptional regulator of BiP. RUNX1 modulates the PERK/eIF2α signaling through BiP, conferring selective advantage for tumor cells under stress.

### Combination with RUNX1 inhibitor could mitigate gemcitabine resistance and inhibit tumor growth

**Fig. 7:**
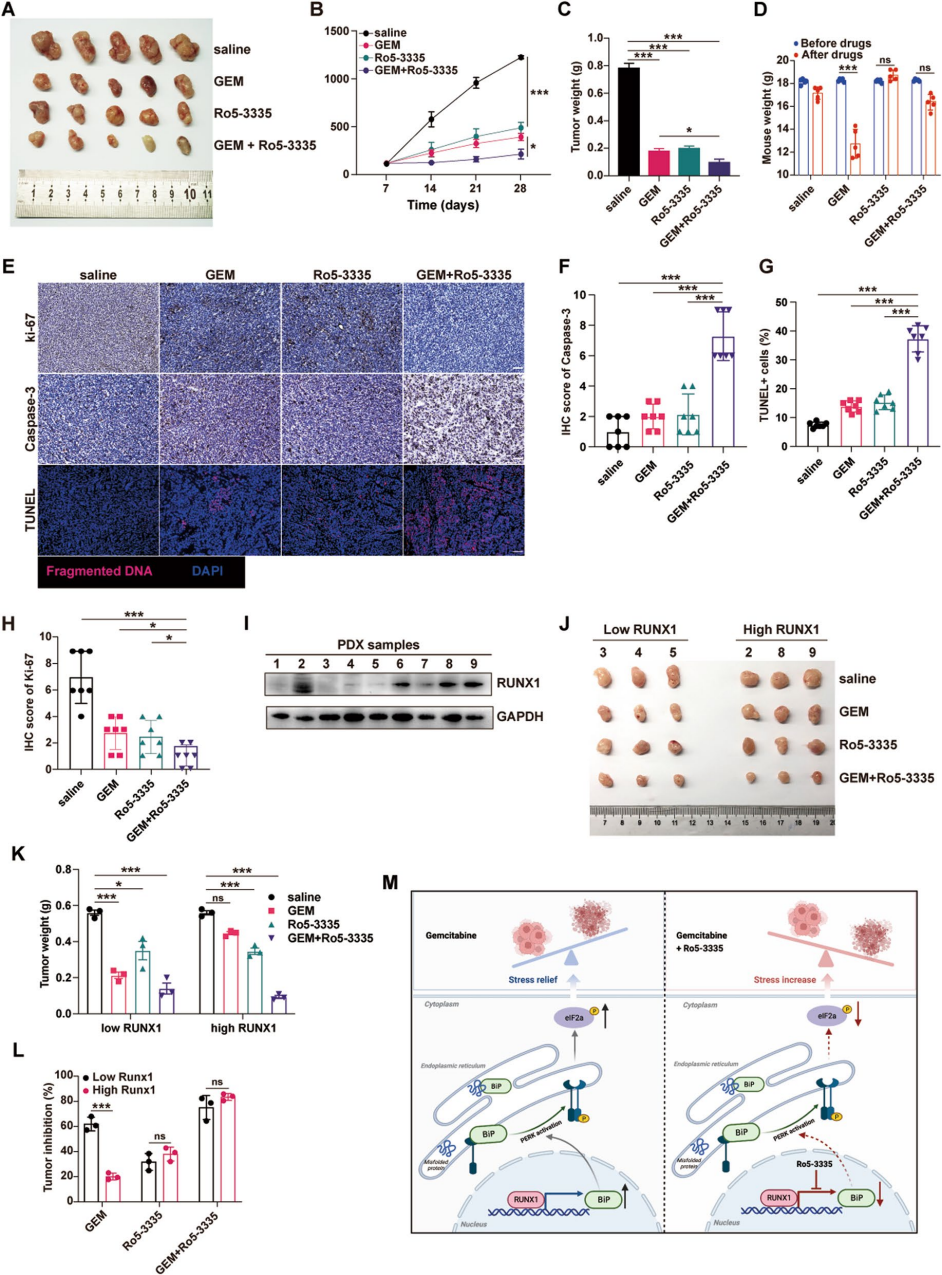
Targeting RUNX1 could reverse gemcitabine resistance and sensitize PDAC to gemcitabine. **A**-**D** Human pancreatic cancer cell line L3.7–2 was subcutaneously transplanted into nude mice, and the other three groups of mice were administered gemcitabine alone, Ro5-3335 alone, or a combination of gemcitabine and Ro5-3335. The tumors were obtained at the end of the experiment. Tumor volumes (**A**, **B**), tumor weight (**C**), and mouse weight (**D**) were analyzed. **E**–**H** The tumors were sliced and stained with Ki-67 and Caspases3. TUNEL kit was used to determine the cell mortality rate. Representative images of Ki-67, Caspases3 staining and TUNEL staining are shown. The IHC scores of BiP, p-eIF2α staining and apoptotic cells rates of each group were evaluated and showed in the column diagram respectively. **I**–**J** Protein levels of RUNX1 in nine PDX were detected by western blotting (**I**). Three cases with high RUNX1 expression and three cases with low RUNX1 expression were used to develop PDX models in NSG mice (*n* = 4 for each case). The mice were treated with saline, gemcitabine alone, Ro5-3335 alone, or a combination of gemcitabine and Ro5-3335. After two weeks, the tumor was removed, and the tumor weight was analyzed. The representative images are shown in (**J**). **K**-**L** Tumor weight and tumor inhibition of the high RUNX1 expression group and low RUNX1 expression group with different treatment are shown. **M** Diagram of RUNX1 facilitates ER stress-mediated GEM-resistance. RUNX1 inhibitor Ro5-3335 displayed an enhanced effect to overcome the GEM-resistance in PDAC. Student’s t-test and ANOVA test were used in the column diagram; scale bar, 100 µm. *, *p* < 0.05; **, *p* < 0.01; ***, *p* < 0.001; ns, no significance

The RUNX1 inhibitor Ro5-3335 exhibited promising effects in various diseases [26], however, its use in PDAC has rarely been reported. To explore the potential value of this RUNX1 inhibitor in PDAC, a subcutaneous xenograft model using human L3.7–2 cells was established, and the effect of Ro5-3335 on tumor growth was evaluated. One week after implantation, mice were intraperitoneally injected with saline control, gemcitabine alone, Ro5-3335 alone, or a combination of Ro5-3335 and gemcitabine at the indicated time points (Supplemental Fig. 6A). Tumor volume was monitored 7 days after implantation by measuring tumor dimensions. Both gemcitabine and Ro5-3335 alone inhibited tumor growth compared to the control. The combination group showed significant inhibition of tumor growth during the entire experimental period (Fig. 7A-B). Consistent with this, tumor weight was significantly reduced in the group receiving combination therapy at the end compared to the control (Fig. 7C). The ITR was also calculated according to tumor weight in each group: 71.1% for Ro5-3335, 76.9% for gemcitabine, and 87.9% for the combination. Body weight was used as an indicator of nutrient intake. Tumor-bearing mice receiving gemcitabine alone showed a significant decrease in body weight compared to the control, whereas Ro5-3335 alone did not cause weight loss, and the combination of gemcitabine and Ro5-3335 exhibited slight but not significantly different weight loss (Fig. 7D). Tumors treated with the combination of gemcitabine and Ro5-3335 showed decreased Ki-67 and increased Caspase3 activity. Additionally, the TUNEL assay indicated a significant difference in the number of apoptotic cells in the combination group compared to the other groups (Fig. 7E-H).

These treatments were repeated in a subcutaneous xenograft model using gemcitabine-resistant cells BxPC3-GR. The results showed that the ITR was 73.9% in mice receiving Ro5-3335, 38.9% in those receiving gemcitabine, and 83.3% in those receiving the combination. In addition, a significant decrease in body weight was observed in tumor-bearing mice receiving gemcitabine alone compared to the control, whereas Ro5-3335 alone did not cause weight loss, and a small but not significantly lower weight loss was observed in the combination of gemcitabine and Ro5-3335 (Supplemental Fig. 6B-E). These results showed that Ro5-3335 displayed a safe and synergistic effect in inhibiting PDAC tumor growth.

To further confirm the effect of targeting RUNX1 chemically on the gemcitabine response in PDAC, a subcutaneous PDX model was established in NSG mice. Based on the expression of RUNX1 in tumor tissues, the mice were divided into two groups: low RUNX1 (RUNX1^low^) and high RUNX1(RUNX1^high^) (Fig. 7I). The mice were then treated with saline control, gemcitabine alone, Ro5-3335 alone, or a combination of Ro5-3335 and gemcitabine at the indicated times. After three weeks, the mice were sacrificed to obtain tumors. In mice receiving the combined treatment of gemcitabine and Ro5-3335, the reduction in tumor weight was significant compared to that in the control group, and ITR was close to 80% in both groups. In mice receiving Ro5-3335 alone, the tumor weight was reduced, but to a limited extent. Also observed was a significant decrease in the RUNX1^low^ group (ITR 60%) treated with gemcitabine (Fig. 7J-L) but a small, insignificant reduction was seen in the RUNX1^high^ group (ITR 18%). Thus, targeting RUNX1 may be effective in inhibiting tumor growth and enhancing the efficacy of gemcitabine therapy in PDAC, even for the GEM-resistant ones.

## Discussion

Although gemcitabine-based chemotherapy is the mainstay therapy for advanced and metastatic pancreatic cancer, the development of chemoresistance severely limits treatment efficiency. In contrast, resistance to gemcitabine is more common in clinics [27].

ER stress plays a critical role in the development of gemcitabine resistance in PDAC [28, 29]; however, factors modulating ER stress have rarely been reported. RUNX1 is involved in cell differentiation, lineage destination and development of organs [30]. It is not only the causative factor for malignant blood diseases but also plays a pro-cancer or inhibitory role in solid malignancies. Though RUNX1 was identified the oncogenic role in tumor proliferation and distant metastases, and also a prognostic marker for PDAC owing to it related to shorter survival [17, 31]; however, the versatile roles of RUNX1 in PDAC are not well known. It is worth more noting that RUNX1 correlated with ER stress signaling during neurofibromagenesis in recent study [13], but the mechanism behind the modulation of ER stress by RUNX1 has not been fully elucidated. In this study, RUNX1 was identified as a potential GEM-resistant related gene by the differential expression genes (DEGs) analysis, using our scRNA-seq data for PDAC. It was found that RUNX1 promoted cell proliferation and reduced apoptosis induced by gemcitabine, thereby facilitating GEM resistance in PDAC cells. Furthermore, based on the scRNA-seq data and subsequent experiments, itwas determined that RUNX1 functioned by modulating ER stress via the BiP/PERK/eIF2α pathway. Importantly, the RUNX1 inhibitor displayed promising effect in the PDX mouse models when combined with GEM treatment, suggesting that RUNX1 inhibition could be an effective combination therapy for overcoming GEM resistance (Fig. 7M).

RUNX1 is primarily involved in normal hematopoiesis, and its mutation and gene translocation are critical causative factors for leukemia and other malignancy hematological diseases [10]. However, several studies have shown that normal RUNX1 expression is required for the survival of certain types of leukemia cells [32]. This indicates that RUNX1 has a dual effect on hematological diseases, depending on the species. In addition, the same behavior has been observed in cancers. Recent studies have determined that RUNX1 presents somatic mutations such as nonsense, frameshift, and missense mutations, leading to its dysfunction and playing a suppressive role in luminal subtype breast cancer with ER positivity [33, 34]. Nevertheless, significant RUNX1 overexpression in triple negative is correlated with poor outcomes [35]. In PDAC, RUNX1 has been identified as an oncogene in tumor growth and metastasis [14, 17] and the other roles and mechanisms of RUNX1 leading to the malignant progression of PDAC are not well known. In this study, the data showed that RUNX1 correlated with the malignant clinical characteristics of PDAC and portended poor survival, and that RUNX1 enhanced cell proliferation and reduced cell apoptosis induced by gemcitabine, leading to insensitivity to gemcitabine. This suggested the possibility of targeting RUNX1 to overcome gemcitabine resistance in PDAC cells.

Of note is that this study found that RUNX1 modulated the ER stress by the BiP/PERK/eIF2α pathway. The ER stress is determined to confer chemoresistance by involving a wide array of fundamental cellular processes [6, 7]. ER stress has been implicated in gemcitabine resistance through the coordination of fatty acid biosynthesis pathways [29]. Moreover, gemcitabine induced eIF2α phosphorylation and then activated the integrated stress response (ISR), a cytoprotective pathway in pancreatic cancer [20]. Phosphorylation of eIF2α by PERK has been shown to be necessary for the growth of larger solid tumors [22, 36, 37]. In this study, RUNX1 was identified as a most potential GEM-resistance related gene by the comparison analysis of the scRNA-seq data and public datasets for PDAC. Furtherly pathway enrichment analysis revealed strong correlation of RUNX1 with ER stress signaling, especially with the PERK/ eIF2α branch. This is consistent with the findings of PERK/ eIF2α signaling in therapy-resistance that have been reported [22]. We inferred the possibility of RUNX1 modulating ER stress through the BiP/PERK/ eIF2α axis that was subsequently confirmed. Morphological changes in response to ER stress in gemcitabine-treated PDAC cells were observed in vitro, including an enlarged vesicular endoplasmic reticulum around the cell nucleus. Also detected was an obvious increase of ER stress related markers including BiP expression and dominant activity of PERK/eIF2α signaling after gemcitabine treatment, and which were regulated by RUNX1 alteration. Additionally, ER stress and PERK inhibitors were used to confirm the effects of the PERK/eIF2α axis on RUNX1-inducing GEM-resistance. The positive results indicate the protective effect of RUNX1 depending on the activity of PERK/eIF2α axis.

More importantly, the data demonstrates that RUNX1 regulates BiP expression in PDAC. It was reported that RUNX1 correlated with ER stress through transcriptionally activation of ribosome gene expression and increased protein synthesis [13]. However, the mediating molecules during the modulation procedure have not been revealed yet. By the DEGs analysis in our study, BiP was the most critical candidate for RUNX1 regulation of PERK/ eIF2α signaling. BiP is essential for reducing the accumulation of unfolded proteins, impeding the aggravation of ER stress and apoptosis, and enhancing the endurance of cells under stressful conditions [38]. Though BiP generally functions as ER-located chaperon, it plays a transcriptional role in different cellular process through translocation to nucleus [25, 39]. Further, we found RUNX1 could directly bind to the BiP promoter and activate a transcriptional surge. However, elevated BiP expression and p-PERK, but not PERK protein, was found as result of RUNX1 transcriptionally activation in this study. Perhaps it is attributed to the isoform of BiP protein, GRP78va. GRP78va was identified to antagonize the PERK inhibitor P58(IPK) which would terminate PERK activation by inhibiting its kinase activity, in the late phase of the UPR [25, 40]. Additionally, the effects of RUNX1 on cell proliferation and apoptosis were reversed by BiP silencing. it was supposed that RUNX1 transcriptionally increases BiP expression, which leads more PERK proteins to be released and phosphorylated to activate its downstream effector eIF2α, and then reprogrammed the global translation to attenuate the burden of mis-folded or unfolded proteins. This adaptive ER stress thus facilitates acquisition of GEM resistance.

Interestingly, RUNX1 inhibition displayed enhanced effect in PDX mouse models of PDAC. Recently, RUNX1 has been identified as an effective target in various diseases. Some small-molecule inhibitors such as Ro5-3335, AI-4–57, and AI-10–49, showed great therapeutic effect, targeting the interaction of RUNX1 and its cofactor CBFB. Ro5-3335 [15], a lipophilic small-molecule RUNX1 inhibitor belonging to the benzodiazepine family, is safe in animals and exhibits tolerable marrow toxicity [26]. Ro5-3335 synergizes with the histone deacetylase inhibitor SAHA (vorinostat), resulting in reactivation of latent HIV-1 and clearance of HIV-1 [41]. Intravitreal injection of Ro5-3335 significantly decreased the choroidal neovascularization (CNV) area after laser injury [42]. It was also more effective in reducing vascular leakage when combined with anti-VEGF drugs, thus providing a path for patients with neovascular age-related macular degeneration. In a study on proliferative vitreoretinopathy (PVR), Ro5‐3335 was formulated into a nano-emulsion and administered in rabbit PVR models, resulting in the inhibition of disease progression [43]. Moreover, the concentration of this inhibitor was detected at 2.67 ng/mL in the vitreous cavity by mass spectrometry, suggesting the feasibility of targeting RUNX1 for the treatment of this disease. RUNX1 is also of great value in tumor therapy. The combination of Ro5-3335 and cisplatin showed synergistic effects on ovarian cancer cell apoptosis [44]. However, the use of the RUNX1 inhibitor Ro5-3335 in PDAC has rarely been reported. In this study, Ro5-3335 in RUNX1^low^ and RUNX1^high^ PDX mouse models were used in combination with gemcitabine. It was observed that Ro5-3335 alone displayed antitumor growth to some extent, although there was no significant difference compared to gemcitabine alone. The combination of gemcitabine and Ro5-3335 was more effective at inhibiting tumor growth in both RUNX1^low^ and RUNX1^high^ PDX mouse models. Additionally, this combined treatment showed effective tumor suppression in the xenografts constructed with GEM-resistant PDAC cells. Moreover, the addition of Ro5-3335 to gemcitabine did not weaken the nutritional status of the mice. It should be mentioned that pharmacological inhibition of RUNX1 in PDAC by AI-10–49 led to transcriptional activation of NOXA, a proapoptotic sensor, promoting NOXA-dependent apoptosis and resulting in synthetic lethality [45].Therefore, targeting RUNX1 may be an attractive option for GEM-resistant PDAC.

## Conclusions

In summary, this study is the first to link RUNX1 expression with gemcitabine resistance in PDAC. It is confirmed that RUNX1 is closely associated with malignant features and poor survival. RUNX1 enhances cell proliferation and reduces apoptosis induced by gemcitabine. Moreover, a correlation between RUNX1 and ER stress was found by scRNA-seq data analysis and it is confirmed that RUNX1 modulates ER stress through activation of the BiP/PERK/eIF2α axis. Furthermore, it is determined that RUNX1 directly binds to the BiP promoter and activates BiP expression, which is primary sensor of ER stress and triggers downstream signaling. More importantly, the RUNX1 inhibitor displayed a safe and promising effect in inhibiting tumor growth in GEM-resistant xenograft and PDX mouse models. This work highlights that combination with RUNX inhibition is a promising therapy to overcome insensitivity to gemcitabine in PDAC. However, most studies on the RUNX1 inhibitor Ro5-3335 have been limited in PDX models, and more preclinical studies should be conducted to achieve its clinical utility.

### Supplementary Information

**Additional file 1:** **Supplemental Table1.** Primers used in the experiments.

**Additional file 2:** **Supplemental Figure 1.** Related to Figure 1. The mRNA expression of RUNX family members in PDAC tissues.

**Additional file 3:** **Supplemental Figure 2.** Related to Figure 2.RUNX1 in vitro facilitates the gemcitabine resistance in PDAC.

**Additional file 4:** **Supplemental Figure 3.** Related to Figure 4.Bioinformatic analysis of association between RUNX1 and ER stress pathway in PDAC at the single-cell level.

**Additional file 5:** **Supplemental Figure 4. **Related to Figure 5. RUNX1 imparts gemcitabine resistance in PDAC through ER stress.

**Additional file 6:** **Supplemental Figure 5. **Related to Figure 6. BiP is necessary for RUNX1-inducing gemcitabine resistance in the PDAC cells.

**Additional file 7:** **Supplemental Figure 6. **Related to Figure 7. Ro5-3335 displays a safe and enhanced effect with gemcitabine in PDAC.

**Additional file 8.** Clinical drug file of patients for PDAC from TCGA dataset.

## Data Availability

HRA000433 (14 patients with PDAC) was downloaded from The Genome Sequence Archive for Human (GSA-Human). CRA001160 (24 patients with PDAC and 11 control pancreatic tissues) was obtained from the Genome Sequence Archive under project PRJCA001063. TCGA RNA-seq data of PDAC tissues were downloaded from the Genomic Data Commons (GDC) portal, and clinical data were obtained from the TCGA Data Portal.

## Abbreviations

BiP: Binding immunoglobulin protein
ChIP: Chromatin immunoprecipitation
CNV: Choroidal neovascularization
CR: Complete response
DEGs: Differentially expressed genes
ER: Endoplasm reticulum
GEM: Gemcitabine
IHC: Immunohistochemistry
IRE1α: The inositol-requiring protein 1α
ISR: The integrated stress response
ITR: Rate of tumor inhibition
PCA: Principal component analysis
PD: Progressive disease
PDAC: Pancreatic ductal adenocarcinoma
PDX: Patient-derived xenograft
PERK: PKR-like endoplasmic reticulum kinase
PVR: Proliferative vitreoretinopathy
scRNA-seq: Single-cell RNA sequencing
TCGA: The Cancer Genome Atlas
UPR: Unfolded protein response
