# Supplementary material for: Combination of RUNX1 inhibitor and gemcitabine mitigates chemo‐resistance in pancreatic ductal adenocarcinoma by modulating BiP/PERK/eIF2α-axis-mediated endoplasmic reticulum stress
Source: J Exp Clin Cancer Res. 2023 Sep 11;42:238. doi: 10.1186/s13046-023-02814-x (PMC10494371; doi:10.1186/s13046-023-02814-x)
Supplement: Supplementary file 1 — Additional file 1: Supplemental Table1. Primers used in the experiments. [file 13046_2023_2814_MOESM1_ESM.docx]

Supplemental Table1 Primers used in the experiments.

| **PCR primers** |  |
| --- | --- |
| Human RUNX1 |  |
| Forward primer | CCTCAGGTTTGTCGGTCGAA |
| Reverse primer | TGCCGATGTCTTCGAGGTTC |
| Human RUNX2 |  |
| Forward primer | GAACCCAGAAGGCACAGACA |
| Reverse primer | ACTTGGTGCAGAGTTCAGGG |
| Human RUNX3 |  |
| Forward primer | ACCTTCATCCGCGACCCAAGC |
| Reverse primer | CGCCAGCACGTCCACCATCGAG |
| Human BiP |  |
| Forward primer | CGTCCTATGTCGCCTTCACTCC |
| Reverse primer | TTCTCCCCCTCCCTCTTATCC |
| Human ACTB |  |
| Forward primer | CTACCTTCAACTCCATCATGAAGTG |
| Reverse primer | TGCGCTCAGGAGGAGC |
| Human hRPL2 |  |
| Forward primer | GTTCATGCTGGGAACATCATTGC |
| Reverse primer | TCCACAGCCTCCGTGTTTCTG |
| **ChIP primers** |  |
| BiP binding motif1 primer | |
| Forward primer | TTGTCCTGCGACTTACCGTG |
| Reverse primer | CCAGCTTCCATCCAGTCTC |
| BiP binding motif2 primer | |
| Forward primer | CTGCTGGACCTACTCCGAC |
| Reverse primer | ATTCATTGGCTGCTATTCGTT |
