# Supplementary figures and images for: Combination of RUNX1 inhibitor and gemcitabine mitigates chemo‐resistance in pancreatic ductal adenocarcinoma by modulating BiP/PERK/eIF2α-axis-mediated endoplasmic reticulum stress
Source: J Exp Clin Cancer Res. 2023 Sep 11;42:238. doi: 10.1186/s13046-023-02814-x (PMC10494371; doi:10.1186/s13046-023-02814-x)

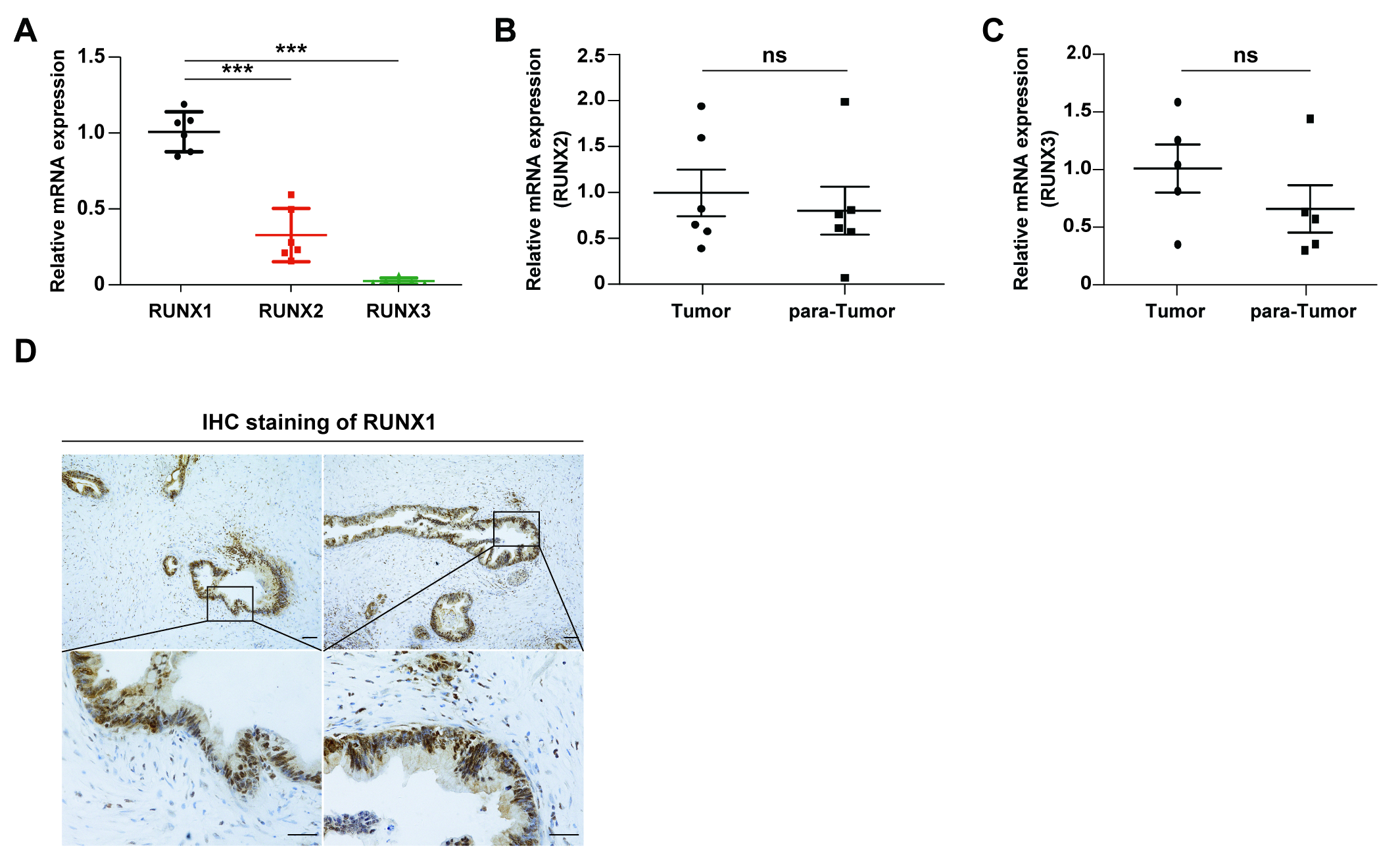

Supplement: Supplementary file 2 — Additional file 2: Supplemental Figure 1. Related to Figure 1. The mRNA expression of RUNX family members in PDAC tissues. [file 13046_2023_2814_MOESM2_ESM.tif]

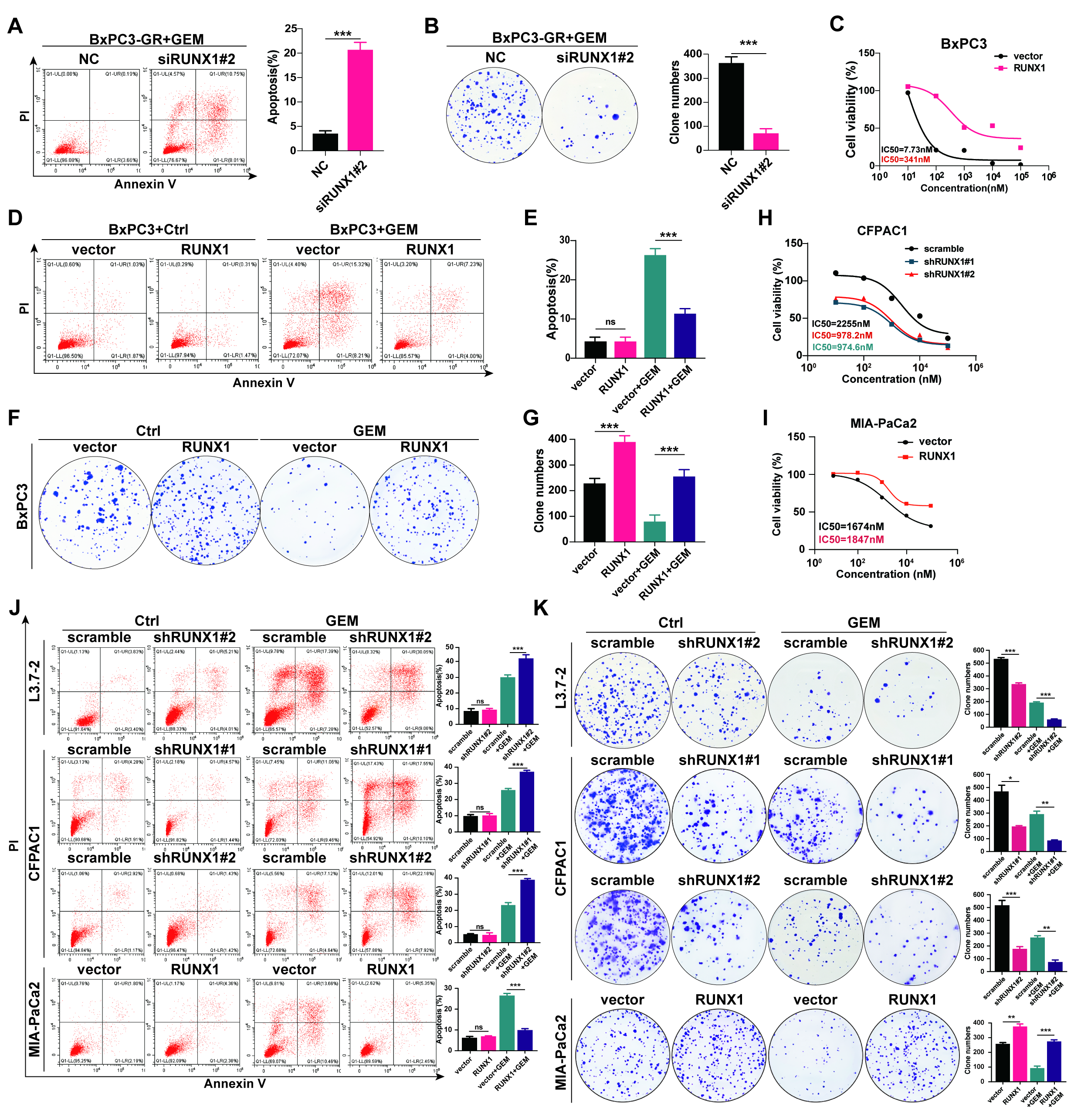

Supplement: Supplementary file 3 — Additional file 3: Supplemental Figure 2. Related to Figure 2.RUNX1 in vitro facilitates the gemcitabine resistance in PDAC. [file 13046_2023_2814_MOESM3_ESM.tif]

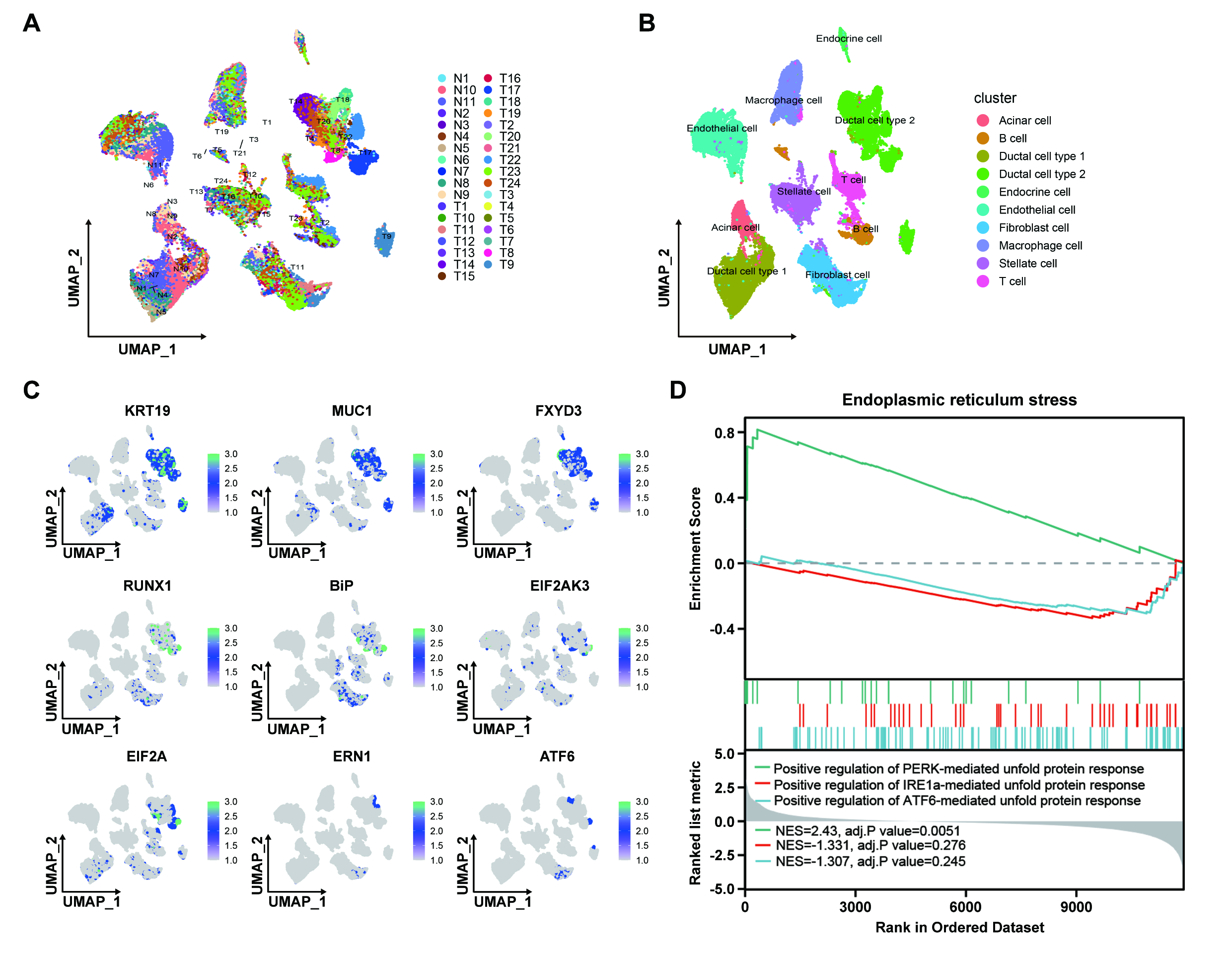

Supplement: Supplementary file 4 — Additional file 4: Supplemental Figure 3. Related to Figure 4.Bioinformatic analysis of association between RUNX1 and ER stress pathway in PDAC at the single-cell level. [file 13046_2023_2814_MOESM4_ESM.tif]

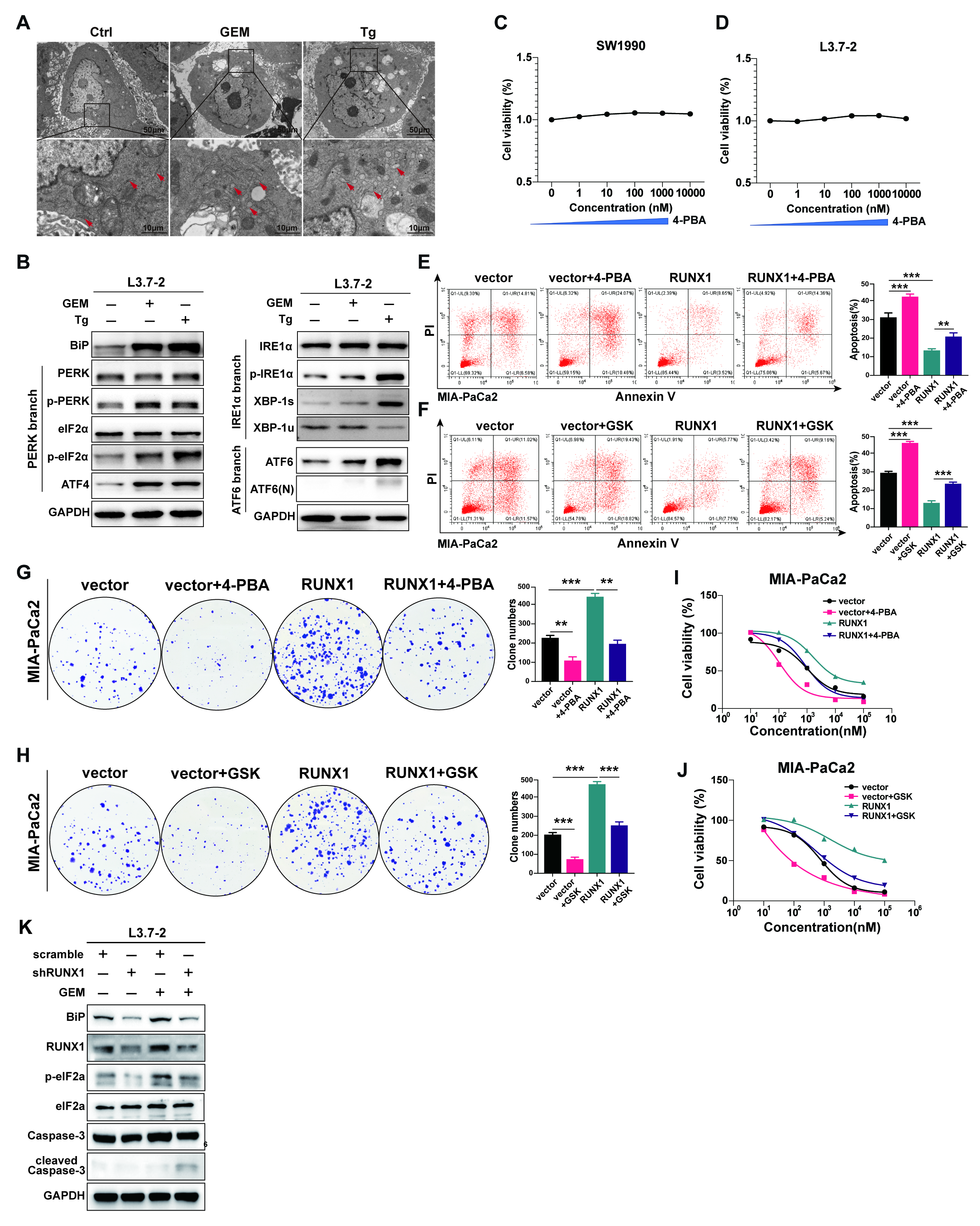

Supplement: Supplementary file 5 — Additional file 5: Supplemental Figure 4. Related to Figure 5. RUNX1 imparts gemcitabine resistance in PDAC through ER stress. [file 13046_2023_2814_MOESM5_ESM.tif]

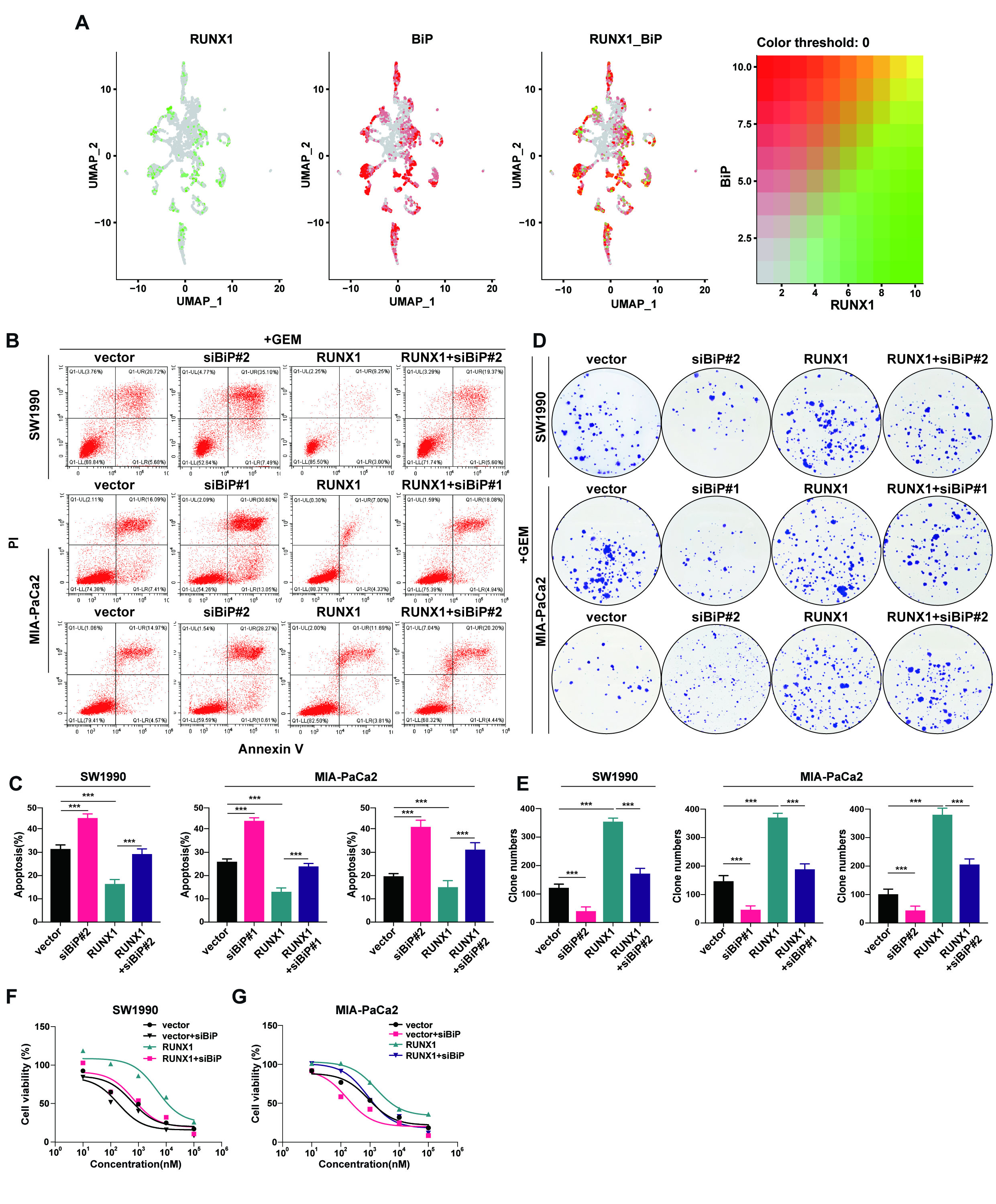

Supplement: Supplementary file 6 — Additional file 6: Supplemental Figure 5. Related to Figure 6. BiP is necessary for RUNX1-inducing gemcitabine resistance in the PDAC cells. [file 13046_2023_2814_MOESM6_ESM.tif]

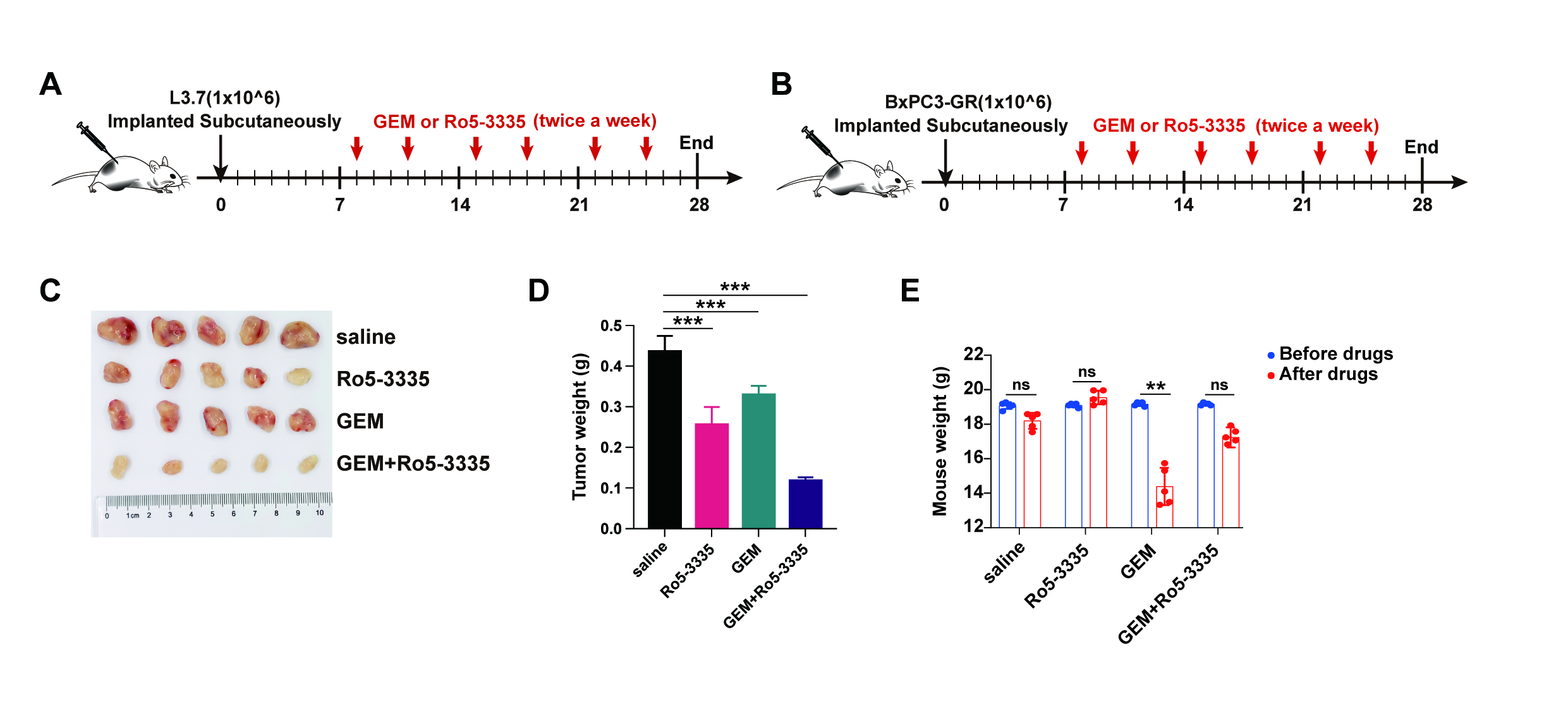

Supplement: Supplementary file 7 — Additional file 7: Supplemental Figure 6. Related to Figure 7. Ro5-3335 displays a safe and enhanced effect with gemcitabine in PDAC. [file 13046_2023_2814_MOESM7_ESM.tif]
